# AhR-Siglec-15 axis regulates lysosomal Ca^2+^ release for sonic hedgehog medulloblastoma growth via TRPML1

**DOI:** 10.1093/procel/pwaf100

**Published:** 2025-11-19

**Authors:** Zhenfeng Wang, Shunshun Li, Xu Sun, Dianheng Wang, Yabo Zhou, Li Zhou, Jie Chen, NanNan Zhou, Qiuying Zhu, Jinyan Liu, Guihong Zhang, Wei-Min Tong, Jiadi Lv, Bo Huang

**Affiliations:** Department of Immunology, State Key Laboratory of Common Mechanism Research for Major Diseases, Institute of Basic Medical Sciences and School of Basic Medicine, Chinese Academy of Medical Sciences (CAMS) and Peking Union Medical College, Beijing 100005, China; Department of Immunology, Basic Medicine College, China Medical University, Shenyang 110122, China; Department of Laboratory Medicine, National Clinical Research Center for Laboratory Medicine, The First Hospital of China Medical University, Shenyang 110001, China; Department of Immunology, State Key Laboratory of Common Mechanism Research for Major Diseases, Institute of Basic Medical Sciences and School of Basic Medicine, Chinese Academy of Medical Sciences (CAMS) and Peking Union Medical College, Beijing 100005, China; Department of Immunology, State Key Laboratory of Common Mechanism Research for Major Diseases, Institute of Basic Medical Sciences and School of Basic Medicine, Chinese Academy of Medical Sciences (CAMS) and Peking Union Medical College, Beijing 100005, China; Department of Immunology, State Key Laboratory of Common Mechanism Research for Major Diseases, Institute of Basic Medical Sciences and School of Basic Medicine, Chinese Academy of Medical Sciences (CAMS) and Peking Union Medical College, Beijing 100005, China; Department of Immunology, State Key Laboratory of Common Mechanism Research for Major Diseases, Institute of Basic Medical Sciences and School of Basic Medicine, Chinese Academy of Medical Sciences (CAMS) and Peking Union Medical College, Beijing 100005, China; Department of Immunology, State Key Laboratory of Common Mechanism Research for Major Diseases, Institute of Basic Medical Sciences and School of Basic Medicine, Chinese Academy of Medical Sciences (CAMS) and Peking Union Medical College, Beijing 100005, China; Department of Immunology, State Key Laboratory of Common Mechanism Research for Major Diseases, Institute of Basic Medical Sciences and School of Basic Medicine, Chinese Academy of Medical Sciences (CAMS) and Peking Union Medical College, Beijing 100005, China; Department of Clinical Medicine, Yanjing Medical College, Capital Medical University, Beijing 100069, China; Biotherapy Center and Cancer Center, The First Affiliated Hospital of Zhengzhou University, Zhengzhou 450052, China; Department of Pathology, Pathology Center, Anhui Medical University, Hefei 230032, China; Department of Pathology, Institute of Basic Medical Sciences, CAMS and Peking Union Medical College, Beijing 100005, China; Department of Immunology, State Key Laboratory of Common Mechanism Research for Major Diseases, Institute of Basic Medical Sciences and School of Basic Medicine, Chinese Academy of Medical Sciences (CAMS) and Peking Union Medical College, Beijing 100005, China; Department of Immunology, State Key Laboratory of Common Mechanism Research for Major Diseases, Institute of Basic Medical Sciences and School of Basic Medicine, Chinese Academy of Medical Sciences (CAMS) and Peking Union Medical College, Beijing 100005, China

**Keywords:** Siglec-15, AhR, TFEB, lysosome, TRPML1

## Abstract

Sonic hedgehog subgroup medulloblastoma (SHH-MB), an aggressive pediatric brain tumor that originates from granule neuron precursors, faces the challenge of poor treatment owing to its unclear molecular mechanisms. Here, we show that sialic acid-binding immunoglobulin-like receptor 15 (Siglec-15), an immunosuppressive membrane protein, is upregulated and mediates SHH-MB growth through its translocation to the lysosomal membrane. We found that SHH-MB cells use the cation-independent mannose 6-phosphate receptor (CI-MPR) to transport Siglec-15 from the trans-Golgi network (TGN) to lysosomes, where Siglec-15 induces lysosomal Ca^2+^ release by interacting with mucolipin TRP cation channel 1 (TRPML1), leading to the nuclear translocation of the transcription factor EB (TFEB). Blockade of Siglec-15, TRPML1, or TFEB hinders SHH-MB growth *in vitro* and *in vivo*. Importantly, aryl hydrocarbon receptor (AhR), a cytoplasmic transcription factor, upregulates Siglec-15 expression. AhR inhibition by CH-223191 or StemRegenin 1 (SR1) achieved therapeutic efficacy against orthotopic SHH-MB xenografts in mice. These findings reveal an essential role for the AhR-Siglec-15 axis in SHH-MB development, providing a potential strategy for SHH-MB treatment.

## Introduction

Medulloblastoma (MB), a neuronally invasive embryonal malignancy, is among the most common cerebellar tumors in children (Northcott et al., 2019), and commonly arises in the vermis and projects into the fourth ventricle (Louis et al., 2016; Millard and De Braganca, 2016). Based on molecular characteristics, MBs have been classified into four subgroups: wingless (WNT), SHH, Group 3, and Group 4 (Louis et al., 2016, 2021; Orr, 2020). In WNT–MB patients, tumorigenesis is driven by activating β-catenin mutations that constitutively stimulate WNT signaling. In contrast, the most common genetic events in Group 3 and Group 4 are high-level amplifications of the MYC oncogene and overexpression of PRDM6, respectively (Northcott et al., 2017). The SHH subgroup, a major subtype that involves sonic hedgehog (SHH) signaling, which is essential for the development of the cerebellum and tumorigenesis, has been extensively investigated (Chen et al., 2022; Garcia-Lopez et al., 2021; Zhang et al., 2022). Patched 1 (PTCH1) is an inhibitor of SHH signaling. During cerebellar development, Purkinje cells secrete SHH, a major mitogen of cerebellar granule cell progenitors, into the external granule cell layer. By binding to the SHH ligand, PTCH1 relieves its inhibition of smoothened (SMO). Activation of SMO triggers downstream signaling events by releasing glioma-associated oncogene (GLI) transcription factors from suppressor of fused (SUFU), a negative regulator of the pathway. This allows GLIs to translocate to the nucleus and induce activation of SHH pathway target genes. The *PTCH1* mutation derepresses SMO and activates GLI for MB formation (Wang et al., 2022; Zhang et al., 2022). Thus, targeting SHH signaling has been highlighted as a critical strategy for SHH-MB treatment. Notwithstanding this, clinical benefits remain limited, suggesting that alternative signaling pathways may also play an important role in SHH-MB tumorigenesis and are urgently required for identification.

Human sialic acid-binding immunoglobulin-like receptor 15 (Siglec-15) is unique among Siglec family members, because it has a minimal intracellular domain but carries a lysine residue in the transmembrane domain that links the adaptor protein DNAX activation protein 12 (DAP12) or DAP10, implying its functioning as an activating signaling molecule (Kang et al., 2020; Macauley et al., 2014; Smith and Bertozzi, 2021). Surprisingly, Siglec-15 has recently been shown to transduce immunosuppressive signals and to function as an immune checkpoint during cancer immunotherapy (Fan et al., 2019; Kang et al., 2020). In addition, Siglec-15 is widely expressed in human cancers and has a tumor-promoting effect (Cao et al., 2021a; Fan et al., 2019, 2021; Liu et al., 2021). Notably, Siglec-15 has been reported to localize to the cytoplasm with unknown functions (Angata et al., 2007). In this study, we found that Siglec-15, transactivated by the aryl hydrocarbon receptor (AhR), is a critical factor for SHH-MB cell growth. Siglec-15 was translocated from the Golgi apparatus to lysosomes via cation-independent mannose 6-phosphate receptor (CI-MPR), where Siglec-15 interacts with mucolipin TRP cation channel 1 (TRPML1) to mediate lysosomal Ca^2+^ release, thereby activating the transcription factor EB (TFEB) and promoting SHH-MB cell growth. These findings reveal an unknown molecular mechanism, by which SHH-MB cell growth is regulated, and identify AhR and Siglec-15 as molecular targets for SHH-MB treatment.

## Results

### Siglec-15 expression is required for SHH-MB growth

**Figure 1.**
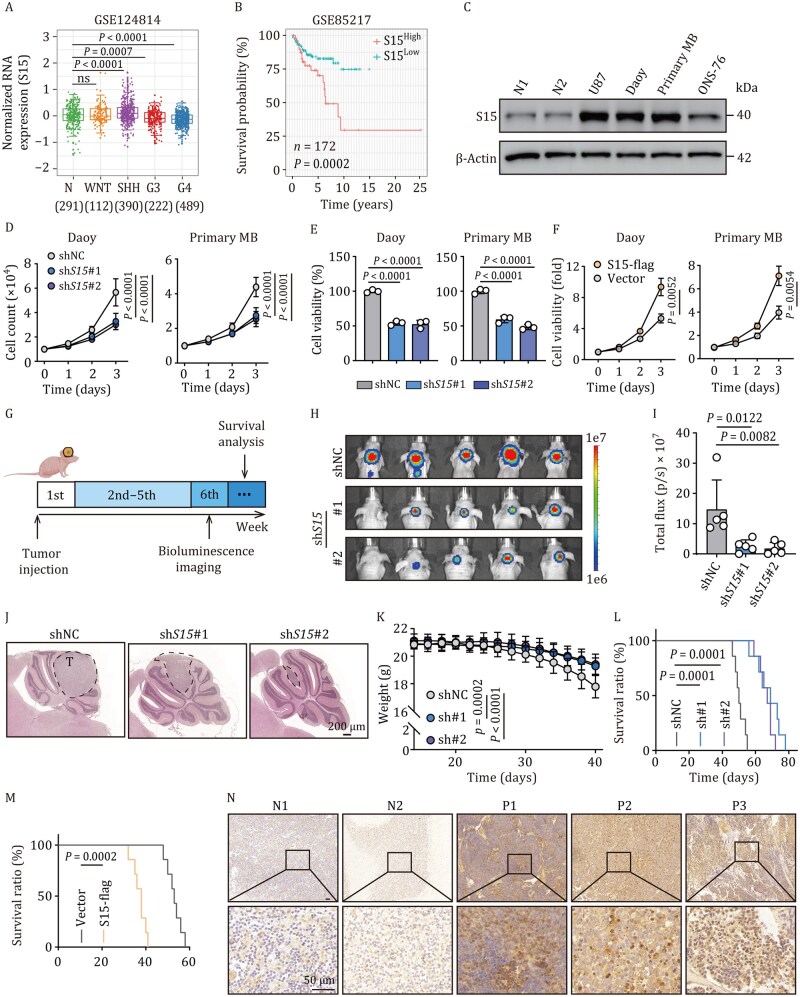
**Siglec-15 promotes SHH-MB growth**. (A) RNA expression profile of Siglec-15 in the four subgroups of MB and normal tissues was analyzed from the GEO database. Data are presented as box plots, *n* = 1,213 for MB tissues and *n* = 291 for normal tissues. S15, Siglec-15. (B) Overall survival analysis of SHH-MB patients (*n* = 172). The optimal cut-point (5.04) for expression data and visualize survival curves was calculated by the R package survminer and survival. (C) Western blot analysis of Siglec-15 expression in normal human primary granule neuron cells, U87, Daoy, primary tumor cells, and ONS-76 cells. (D and E) Cell count (D) and cell viability (E) in shNC and sh*Siglec-15* Daoy and primary tumor cells were analyzed, respectively. (F) Relative cell viability in vector or *Siglec-15*-flag Daoy and primary tumor cells was analyzed. (G) Schematic representations of animal experiments. Firefly luciferase-expressing Daoy cells with Siglec-15 knockdown (5 × 10^5^ cells) were orthotopically injected into mice. (H and I) Tumor formation was obtained by bioluminescence imaging (H), and total flux was analyzed (I) at 6 weeks. NC, negative control, *n *= 5 mice. (J) Mice were sacrificed, and tumor sizes were evaluated by H&E staining. Tumors in tissues were shown outlined by dashed black lines. T, tumor, *n *= 5 mice. Scale bar, 200 µm. (K and L) Mice weight (K) and survival ratio (L) were analyzed; *n *= 5 mice for (K), *n *= 7 mice for (L). (M) The survival ratio of mice injected with vector or *Siglec-15*-flag Daoy cells; *n *= 7 mice. (N) Immunostaining of Siglec-15 in normal human samples and SHH-MB patients’ samples. Scale bar, 50 µm; *n *= 3 independent experiments for (C) and (D–F). One-way ANOVA with Dunnett’s multiple-comparisons test (A, E, and I), two-tailed unpaired Student’s *t*-test (F), two-way ANOVA with Dunnett’s multiple-comparisons test (D and K), or log-rank survival analysis (B, L, and M). The data represent mean ± SD.

Previous studies have integrated 23 transcription datasets into a large MB and normal cerebellar gene expression database (Weishaupt et al., 2019). By analyzing this, we found that the expression of Siglec-15 was lower in the G3 and G4 groups, but was higher in the SHH-MB group than in the healthy control group (Fig. 1A). Using the Group 3 MB cell lines D283 and D341, we found that Siglec-15 was poorly expressed in D283 and D341 cells and Siglec-15 overexpression did not increase their proliferation (Fig. S1A–C). Then, we further used published data (Cavalli et al., 2017) to analyze SHH-MB patient survival and found that Siglec-15 expression was correlated with poor survival (Fig. 1B), suggesting that Siglec-15 may have a SHH-MB-promoting effect. By analyzing the single-cell sequence data of MB patients (Riemondy et al., 2022), we found that Siglec-15 expression in SHH-MB tumor cells was the most abundant, compared to other subgroup MB cells and immune cells (Fig. S1D and S1E). In line with this, Siglec-15 was decently expressed in the Daoy SHH-MB cell line and primary SHH-MB tumor cells isolated from patients with SHH-MB, compared to the U87 glioblastoma cell line, which is known to highly express Siglec-15 (Fan et al., 2019) (Figs. 1C and S1F). By transfecting Siglec-15 shRNA into Daoy and primary SHH-MB tumor cells (Figs. S1G and S1H), we found that cell growth was reduced and cell viability was impaired (Fig. 1D and 1E). In contrast, Siglec-15 overexpression increased cell proliferation (Figs. 1F and S1I). ONS-76 is another SHH-MB cell line that expressed low levels of Siglec-15 (Fig. 1C). As expected, Siglec-15 overexpression promoted ONS-76 cell growth (Fig. S1J and S1K). By determining cell apoptosis and cell cycle in vector- and Siglec-15-overexpressing Daoy cells, we found that Siglec-15 drove cell cycle progression toward mitosis, but had no effect on apoptosis (Fig. S1L and S1M). To validate these results *in vivo*, we constructed Siglec-15 knockdown and firefly luciferase-expressing Daoy cells, and injected them into the cerebella of BALB/c nude mice (Fig. 1G). We found that Siglec-15 knockdown markedly inhibited tumor growth, reduced weight loss, and prolonged mouse survival (Fig. 1H–L). As expected, cerebellar inoculation of Siglec-15-overexpressing Daoy cells accelerated orthotopic tumor growth and shortened the long-term survival of mice (Figs. 1M and S1N–S1P). In addition, immunostaining showed Siglec-15 expression was higher in SHH-MB patient samples than in normal controls (Fig. 1N). Together, these results suggest that Siglec-15 has an SHH-MB-promoting effect.

### Siglec-15 localizes to the lysosomes to mediate Ca^2+^ release

**Figure 2.**
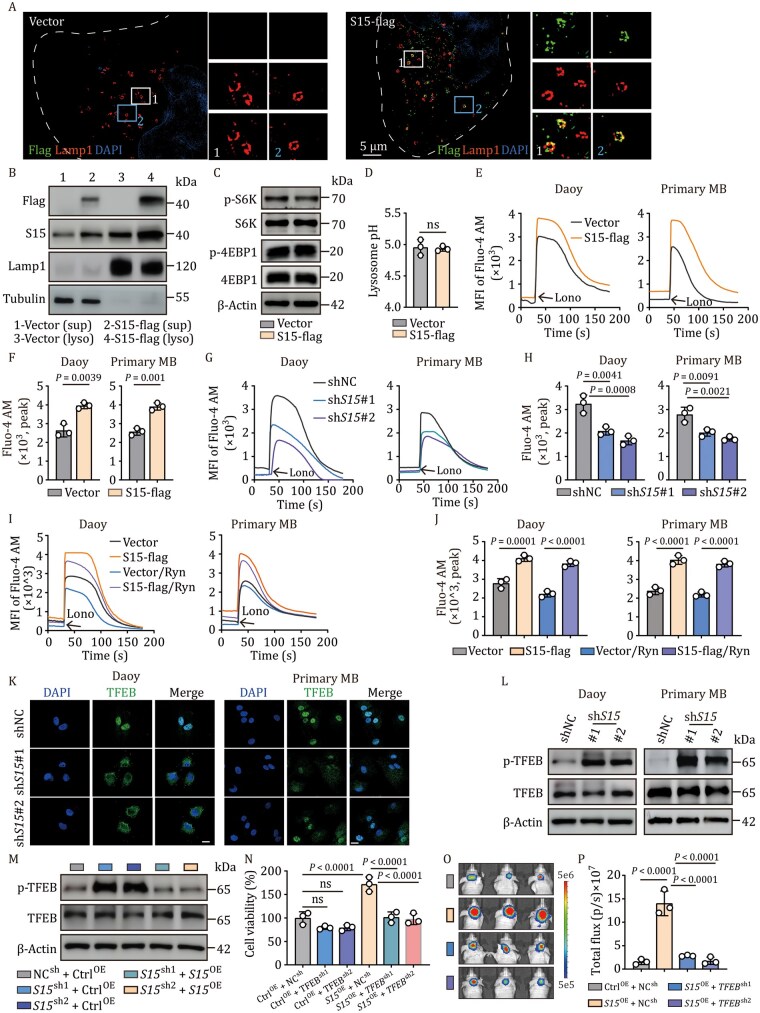
**Siglec-15 mediates lysosomal Ca^2+^ release to activate TFEB**. (A) Immunostaining analysis of *Siglec-15*-flag Daoy cells using lamp1 and flag antibodies. In cellular fluorescence micrographs, representative cells are shown outlined by dashed white lines. Scale bar, 5 µm. (B) The lysosome fraction was prepared, and Siglec-15 was analyzed by Western blot. Sup, supernatant; lyso, lysosome. (C) p-S6K, S6K, p-4EBP1 and 4EBP1 were analyzed by western blot in vector and *Siglec-15*-flag Daoy cells. (D) Lysosomal pH was measured by microplate reader after LysoSensor^™^ Yellow/Blue DND-160 staining in vector and *Siglec-15*-flag Daoy cells. (E–H) *Siglec-15*-flag (E) or sh*Siglec-15* (G) Daoy and primary tumor cells were loaded with Fluo-4 AM, and cytosolic calcium release was recorded by confocal microscope. The peak of Fluo-4 AM was analyzed, respectively (F and H). (I and J) Vector and *Siglec-15*-flag Daoy or primary tumor cells were pretreated with ER calcium release inhibitor ryanodine (10 µmol/L) for 2 h, and cytosolic calcium release was recorded by confocal microscope (I). The peak of Fluo-4 AM was analyzed (J). (K) Immunofluorescence staining of TFEB in sh*Siglec-15* Daoy and primary tumor cells. Scale bar, 20 µm. (L and M) Expression of p-TFEB, total TFEB, and β-actin in Daoy and primary tumor cells transfected with shNC or sh*Siglec-15* or sh*Siglec-15*-*Siglec-15*-flag was detected by western blot. (N) Cell viability in vector, sh*TFEB*, *Siglec-15*-flag, and *Siglec-15*-flag-sh*TFEB* Daoy cells was analyzed. (O and P) Vector, *Siglec-15*-flag and *Siglec-15*-flag-sh*TFEB* Daoy cells (5 × 10^5^ cells) were orthotopically injected into mice. Tumor formation was obtained by bioluminescence imaging (O) and total flux was analyzed (P) at 4 weeks; *n *= 3 mice. In (A–N), *n *= 3 independent experiments. ns, not significant, Two-tailed unpaired Student’s *t*-test (D and F), one-way ANOVA with Dunnett’s multiple-comparisons test (H) or Tukey’s multiple comparisons test (J, N, and P). The data represent mean ± SD.

Next, we investigated the molecular basis of Siglec-15-promoted SHH-MB growth. In addition to the plasma membranes, Siglec-15 has been reported to be present in the cytoplasm (Angata et al., 2007). Using Siglec-15-overexpressing Daoy cells or primary SHH-MB tumor cells for immunostaining, we found that approximately 60% of Siglec-15 localized in the cytoplasm, while the remaining 40% was found on the plasma membrane (Fig. S2A). Ultra-high super-resolution microscopy showed that Siglec-15 did not colocalize with the mitochondria or endoplasmic reticulum (ER) and was slightly colocalized with the endosomes and Golgi apparatus, but mainly with the lysosomes in Daoy and ONS-76 cells (Figs. 2A and S2B–D). Consistently, western blot analysis showed that the majority of Siglec-15 protein molecules were present in the lysosomes (Fig. 2B). This unexpected lysosomal localization prompted us to speculate that Siglec-15 mobilizes lysosomes for tumor promotion. The mTOR signaling pathway is lysosome-dependent (Saxton and Sabatini, 2017) and exerts a tumor-promoting effect. However, transfection with Siglec-15-flag or Siglec-15 knockdown did not alter mTOR activity in Daoy and ONS-76 cells, excluding that Siglec-15 uses mTOR for tumor growth (Figs. 2C, S2E and S2F). Lysosomes are characterized by their acidic pH, which is also involved in tumor progression (Steffan et al., 2010). Again, Siglec-15 did not alter lysosomal pH in Daoy and ONS-76 cells (Figs. 2D and S2G). We then examined lysosomal Ca^2+^ release into the cytosol, which might augment cell proliferation (Cao et al., 2021b). Intriguingly, cytosolic Ca^2+^ levels were elevated in Siglec-15-flag-tranfected Daoy and primary SHH-MB tumor cells, compared to those in control cells (Figs. 2E, 2F, S2H and S2I). Knockdown of Siglec-15 reduced cytosolic Ca^2+^ levels, suggesting that Siglec-15 promotes the release of Ca^2+^ from organelles into the cytosol (Fig. 2G and 2H). In line with this notion, use of ryanodine, which blocks ER Ca^2+^ release, did not affect Siglec-15-increased Ca^2+^ levels in the cytosol (Figs. 2I, 2J, S2J and S2K). Glycyl-L-phenylalanine 2-naphthylamide (GPN) is a widely used lysosomotropic agent that evokes cytosolic Ca^2+^ signals (Moccia et al., 2021). By treating the cells with GPN, we found that the calcium levels in the cells also increased (Fig. S2L and S2M). These results suggest that Siglec-15 localizes to the lysosomes to mediate Ca^2+^ release. TFEB, a master regulator of lysosomal biogenesis, autophagy, and signaling pathways, may be upregulated in tumor cells and is associated with tumor growth and proliferation (Franco-Juárez et al., 2022; Settembre et al., 2011). Notably, lysosome-released Ca^2+^ can activate TFEB effectively (Chen et al., 2018; Wei et al., 2023). In line with this, knockdown of Siglec-15 dampened the translocation of TFEB into the nucleus (Fig. 2K), concomitant with increased TFEB phosphorylation in Daoy and primary SHH-MB tumor cells (Figs. 2L and S2N); however, this effect was rescued by Siglec-15 overexpression (Fig. 2M). By knocking down TFEB (Fig. S2O and S2P), we found that Siglec-15-promoted Daoy cell viability was reduced (Fig. 2N). By injecting TFEB-knockdown and Siglec-15-overexpressing Daoy cells into mice, we found that TFEB knockdown counteracted the promoting effect of Siglec-15 on tumor growth (Fig. 2O and 2P). Together, these results suggest that Siglec-15 localizes to the lysosomes to mediate Ca^2+^ release and subsequent TFEB activation, thus promoting SHH-MB cell growth.

### Siglec-15 interacts with TRPML1 to release lysosomal Ca^2+^

**Figure 3.**
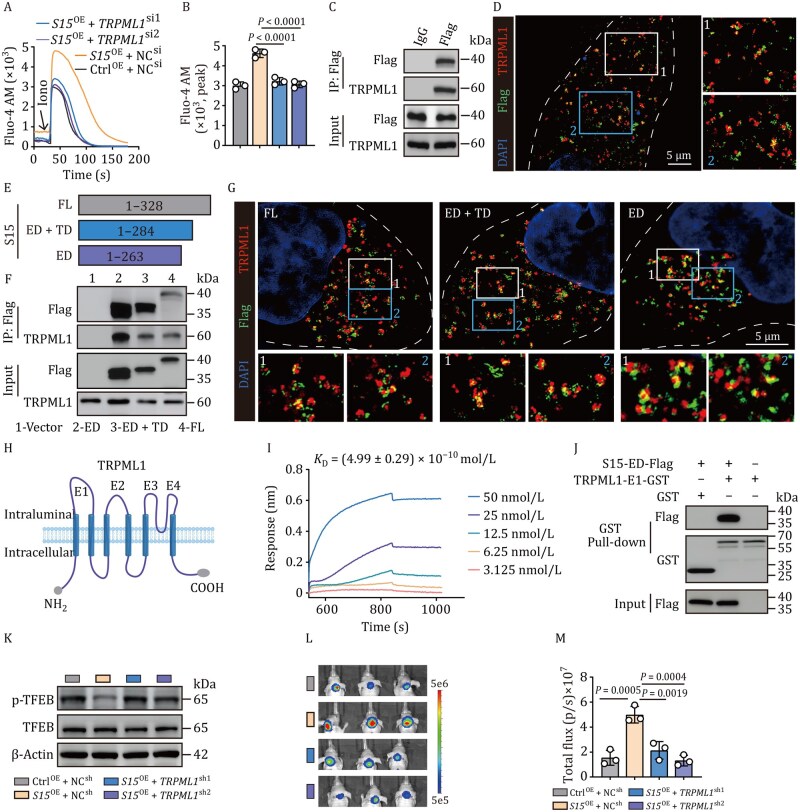
**Siglec-15 interacts with TRPML1 to export lysosomal Ca^2+^ into the cytosol**. (A and B) *Siglec-15*-flag Daoy cells with or without *TRPML1* knockdown were loaded with Fluo-4 AM, and cytosolic calcium release was recorded using a confocal microscope (A). The peak of Fluo-4 AM was analyzed (B). (C) Immunoblot of immunoprecipitation of flag in lysates from *Siglec-15*-flag Daoy cells. (D) Immunostaining analysis of *Siglec-15*-flag Daoy cells using TRPML1 and flag antibodies. Scale bar, 5 µm. (E) Schematic representation of the construction of different motifs found in Siglec-15, as well as the truncated forms used in co-immunoprecipitation assays. FL, full length; ED, extracellular domain; TD, transmembrane domain. (F and G) Interaction between truncated forms of Siglec-15 and TRPML1 was determined by co-immunoprecipitation assays (F) and immunofluorescence staining (G). Scale bar, 5 µm. (H) Schematic representation of TRPML1 subunits. (I) The binding between the recombinant Siglec-15 ED domain and TRPML1 E1 domain was measured by biolayer interferometry. (J) Purified recombinant human TRPML1 E1 domain was incubated with *Siglec-15*-ED-flag 293 T cell lysates for 4 h. Pulldown assays were performed and analyzed by western blot. (K) Expression of p-TFEB, total TFEB, and β-actin in Vector, Siglec-15-flag, and Siglec-15-flag-shTFEB Daoy cells was detected by western blot. (L and M) Vector, Siglec-15-flag and Siglec-15-flag-sh*TRPML1* Daoy cells (5 × 10^5^ cells) were orthotopically injected into mice. Tumor formation was obtained by bioluminescence imaging (L), and total flux was analyzed (M) at 4 weeks. In (A–D, F, G, I, J, and K), *n *= 3 independent experiments. One-way ANOVA with Tukey’s multiple comparisons test (B and M). The data represent mean ± SD.

Next, we explored the manner by which Siglec-15 promoted lysosomal Ca^2+^ release. Lysosomal Ca^2+^ release can be mediated by two-pore channels (TPCs), purinergic receptor X4 (P2X4), and mucolipin subfamily of transient receptor potential channels (TRPMLs) (Cao et al., 2021b; Li et al., 2019). We found that knockdown of TPC1, TPC2, or P2X4 did not alter the effect of Siglec-15 on lysosomal Ca^2+^ release (Fig. S3A–D); however, the knockdown of TRPML1 or inhibiting TRPML1 by ML-SI3 blocked Siglec-15-promoted Ca^2+^ release, suggesting that Siglec-15 may target TRPML1 for lysosomal Ca^2+^ release (Figs. 3A, 3B and S3E–G). Co-immunoprecipitation (Co-IP) showed that TRPML1 was pulled down (Fig. 3C), suggesting an interaction between Siglec-15 and TRPML1. A consistent result was obtained from ultra-high super-resolution microscopy (Fig. 3D). To confirm this interaction, we deleted various domains and constructed different Siglec-15 mutants, in which the original signal peptide was included (Fig. 3E). We found that the deletion of the cytoplasmic domain or transmembrane/cytoplasmic region did not alter the interaction of Siglec-15 with TRPML1 (Fig. 3F and 3G), suggesting the N-terminal extracellular domain (ED) mediates the binding. Given that TRPML1 is a six-transmembrane protein with the N and C termini facing the cytosol (Fig. 3H), we constructed the intraluminal four loops (termed as E1–E4) of TRPML1, respectively. By performing bio-layer interferometry (BLI), we found that the recombinant Siglec-15-N (263 AAs) directly interacted with E1 but not E2, E3, or E4 (Figs. 3I, S3H and S3I). By performing glutathione S-transferase (GST) pull-down assay, we additionally verified that TRPML1 used its E1 domain to interact with Siglec-15 (Figs. 3J, S3J and S3K). In accordance with this, the knockdown of TRPML1 increased TFEB phosphorylation in Daoy cells (Fig. 3K). By injecting TRPML1-knockdown and Siglec-15-overexpressing Daoy cells into mice, we found that TRPML1 knockdown counteracted the promoting effect of Siglec-15 on tumor growth (Fig. 3L and 3M). Together, these results suggest that Siglec-15 binds to TRPML1, thus opening the calcium ion channel for lysosomal Ca^2+^ release.

### CI-MPR mediates Siglec-15 transport from the Golgi to the lysosomes

**Figure 4.**
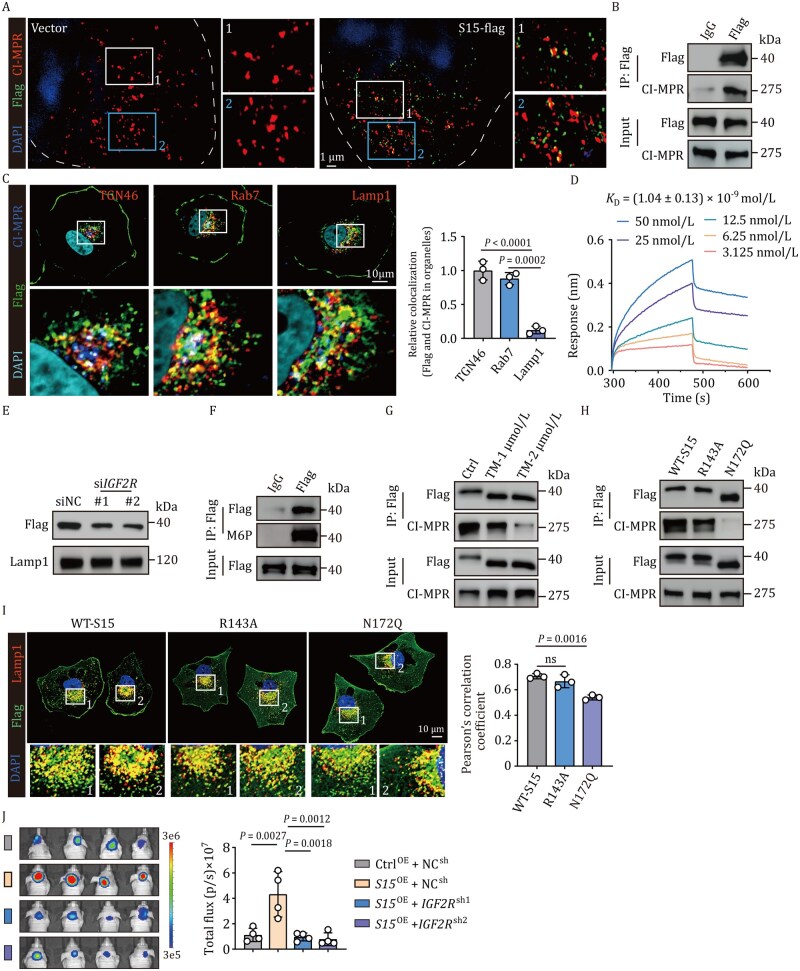
**Siglec-15 is transported into lysosomes by CI-MPR**. (A) Immunostaining analysis of *Siglec-15*-flag Daoy cells using flag and CI-MPR antibodies. Scale bar, 1 µm. (B) Immunoblot of immunoprecipitations of flag in lysates from *Siglec-15*-flag Daoy cells. (C) Immunostaining analysis of *Siglec-15*-flag Daoy cells using flag, CI-MPR, TGN46, rab7, and lamp1 antibodies. TGN46, *trans*-Golgi network marker; Rab7, endosome marker; lamp1, lysosome marker. The white color indicates the co-localization of Siglec-15, CI-MPR, TGN46, or Rab7 (left). Scale bar, 10 µm. Relative colocalization was calculated (right). (D) Binding between CI-MPR and recombinant Siglec-15 ED was measured using biolayer interferometry. (E) Lysosomes were isolated in *Siglec-15*-flag Daoy cells transfected with siNC or si*IGF2R*, and the level of Siglec-15 in lysosomes was analyzed by western blot. (F) Immunoblot of immunoprecipitation of flag in lysates from Siglec-15-flag Daoy cells. (G) Immunoblot of immunoprecipitation of flag in lysates from *Siglec-15*-flag Daoy cells treated with Tunicamycin (1 µmol/L, 2 µmoL/L) for 24 h. (H) Immunoblot of immunoprecipitation of flag in lysates from *Siglec-15*-flag, *Siglec-15*-R143A, or N172Q Daoy cells. (I) Immunostaining analysis of *Siglec-15*-flag, *Siglec-15*-R143A, or N172Q Daoy cells using lamp1 and flag antibodies. Scale bar, 10 µm (left). Pearson’s correlation coefficient between flag and lamp1 (right). (J) Vector, *Siglec-15*-flag and *Siglec-15*-flag-sh*IGF2R* Daoy cells (5 × 10^5^ cells) were orthotopically injected into mice. Tumor formation was obtained by bioluminescence imaging (left) and total flux was analyzed (right); *n *= 4 mice. In (A–I), *n *= 3 independent experiments. ns, not significant, one-way ANOVA with Dunnett’s multiple-comparisons test (I) or Tukey’s multiple comparisons test (J). The data represent mean ± SD.

Siglec-15 is a well-known transmembrane protein that prompted us to explore the pathway through which Siglec-15 is translocated to lysosomes, where it interacts with TRPML1. Endocytosis is a general process that involves translocation of plasma membrane proteins into lysosomes. As shown in Fig. S2B, Siglec-15 colocalized with the endosomes and Golgi apparatus. However, the use of various endocytosis inhibitors did not alter Siglec-15 localization to the lysosomes (Fig. S4A), suggesting that Siglec-15 from the plasma membrane did not contribute to its localization to lysosomes. Considering that Golgi transportation can mediate proteins to localize to lysosomes, we focused on the CI-MPR, a type-I integral membrane glycoprotein that binds to mannose 6-phosphate (M6P)-containing lysosomal proteins and transports them from the Golgi to the endosomal–lysosomal system (Coutinho et al., 2012; Ghosh et al., 2003). Ultra-high super-resolution microscopy analysis and Co-IP showed that CI-MPR and Siglec-15 were co-localized (Fig. 4A and 4B), and such co-localization occurred in the Golgi and endosomes (Fig. 4C). In line with this, BLI showed a direct interaction between Siglec-15 and CI-MPR (Fig. 4D). Low lysosomal pH triggers CI-MPR dissociation with cargo in the lysosomes and allows CI-MPR to recycle to the trans-Golgi network (TGN; Wang et al., 2020). Consistent with this, immunostaining showed minimal colocalization of Siglec-15 and CI-MPR in the lysosomes (Fig. 4C). Blocking this transport route with *CI-MPR* knockdown led to a decrease of Siglec-15 in the lysosomes (Figs. 4E and S4B–D), suggesting that CI-MPR mediates the transport of Siglec-15 from the Golgi to lysosomes. We then dissected the structural basis of the binding of Siglec-15 to CI-MPR. Structurally, Siglec-15 may contain mannose-6-phosphate owing to its N-linked glycosylation (Chen et al., 2020), prompting us to speculate that Siglec-15 binds to CI-MPR via M6P. Using a published method (Pechincha et al., 2022) to detect M6P-containing proteins, we verified that Siglec-15 contained M6P modifications (Fig. 4F). In addition, we found that treating tumor cells with tunicamycin, an inhibitor of N-linked glycosylation (Oliveras et al., 2020), disrupted the association between Siglec-15 and CI-MPR (Figs. 4G and S4E). Consistently, mutation of the Siglec-15 glycosylation site (N172Q) abrogated the interaction of Siglec-15 with CI-MPR in Daoy cells. In contrast, mutation of the sialic acid-binding site (R143A) did not generate a similar result (Fig. 4H). As expected, the N172Q mutation reduced Siglec-15 localization to the lysosomes, the release of Ca^2+^ from lysosomes, and cell viability (Fig. 4I and S4F–S4H). In addition, knockdown of CI-MPR counteracted the promoting effect of Siglec-15 on tumor growth *in vivo* (Fig. 4J). These results suggest that CI-MPR binds Siglec-15 and transports Siglec-15 from the Golgi apparatus to lysosomes.

### Siglec-15 is upregulated by the transcription factor AhR

**Figure 5.**
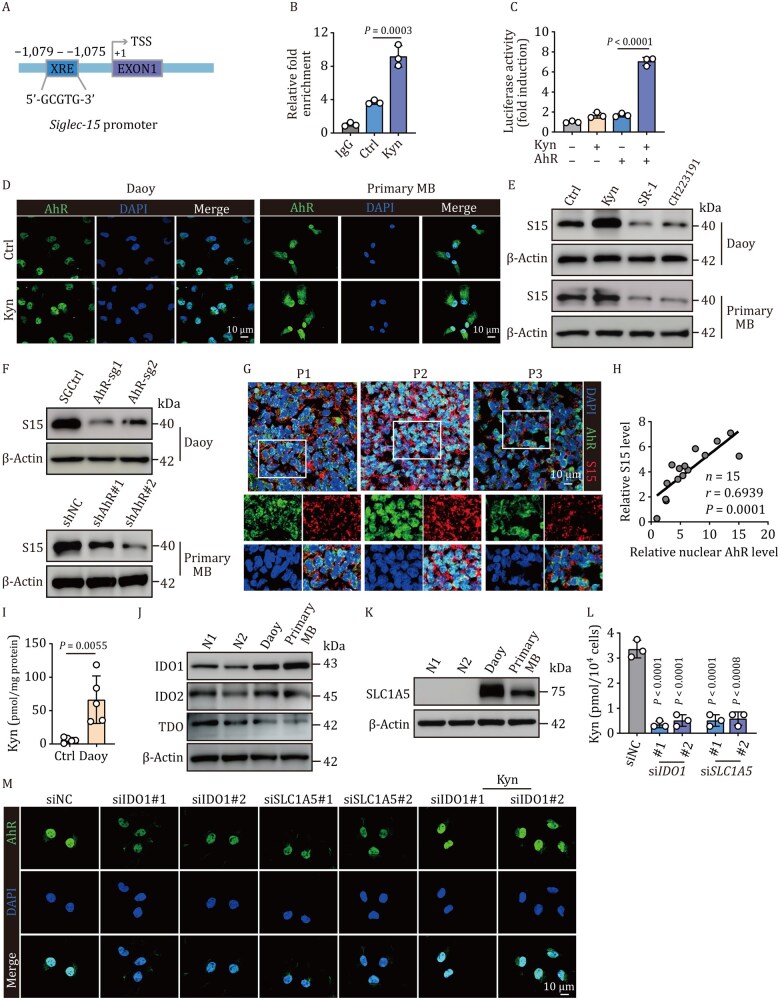
**Siglec-15 expression is regulated by AhR**. (A) Schematic representation of the Siglec-15 promoter region. XRE, xenobiotic response element; TSS, transcription start site. (B) Daoy cells treated with Kyn (400 µmol/L) for 24 h were collected for ChIP–qPCR assay with anti-AhR and specific primers for Siglec-15. (C) 293 T cells were co-transfected with Siglec-15 promoter luciferase reporter PGL4.10 and AhR plasmid for 12 h. Cells were treated with Kyn (400 µmol/L) for another 24 h, followed by analysis of luciferase activity. (D) Immunostaining analysis of AhR in Daoy and primary tumor cells treated with Kyn (400 µmol/L) for 24 h. Scale bar, 10 µm. (E) Expression of Siglec-15 in Daoy and primary tumor cells treated with Kyn (400 µmol/L) or SR-1 (4 µmol/L), or CH223191 (4 µmol/L) for 24 h was determined by western blot. (F) Expression of Siglec-15 in *AhR*-deficient Daoy and primary tumor cells was determined by Western blot. (G and H) Tissue sections from 15 SHH-MB patients were immunohistochemically stained with anti-AhR and anti-Siglec-15 antibodies (G). Relative expression level of Siglec-15 and nuclear AhR in patient samples was shown in (H). Scale bar, 10 µm; *n *= 15 samples. (I) Kyn from control and tumor-bearing mice was measured by high-performance liquid chromatography (HPLC); *n *= 5 mice. (J and K) Western blot analysis of IDO1, IDO2, TDO (J), and SLC1A5 (K) expression in normal human primary granule neuron cells, Daoy, and primary tumor cells isolated from SHH-MB patients. (L) Kyn in siNC, si*IDO1*, or si*SLC1A5* Daoy cells was measured by HPLC. (M) Immunostaining analysis of AhR in siNC, si*SLC1A5*, or si*IDO1* Daoy cells treated with Kyn (400 µmol/L) for 24 h. Scale bar, 10 µm. In (B–F, J–M), *n *= 3 independent experiments. One-way ANOVA with Tukey’s multiple comparisons test (B and C), Dunnett’s multiple-comparisons test (L), two-tailed unpaired Student’s *t*-test (I), or Pearson’s correlation test (H). The data represent mean ± SD.

Next, we investigated the molecular basis of Siglec-15 upregulation in SHH-MB cells. By analyzing the possible transcription factor-binding sites, we found that the xenobiotic response element (XRE) with a core sequence (5′-GCGTG-3′) was present in the promoter region of Siglec-15, which is commonly recognized by AhR (Fig. 5A), a critical cytoplasmic transcription factor that senses xenobiotics and metabolites (Larigot et al., 2022). Previously, we showed that AhR profoundly regulates tumorigenic cell behaviors (Liu et al., 2017, 2018a, 2018b). Coincidentally, AhR has been reported to promote central nervous system tumor development (Opitz et al., 2011; Takenaka et al., 2019), suggesting that Siglec-15 may be regulated by AhR. ChIP-qPCR showed that AhR bound to the Siglec-15 promoter region, and its transactivation was verified by luciferase assay (Fig. 5B and 5C). Kynurenine (Kyn), a typical AhR ligand that is produced from indole-2,3-dioxygenase 1 (IDO1)-catalyzed tryptophan, was found to promote the entry of AhR into the nucleus and upregulate Siglec-15 expression (Figs. 5D, 5E and S5A). As expected, the AhR inhibitor StemRegenin 1 (SR1) or CH-223191 downregulated the expression of Siglec-15 as well as *CYP1A1* and *CYP1B1*, two AhR-targeted genes (Fig. 5E and S5A). AhR knockout also downregulated Siglec-15 expression in Daoy cells (Figs. 5F and S5B–S5D). Similar results were obtained in primary SHH-MB tumor cells (Figs. 5D–F and S5C–E). In addition, we found that AhR levels in the nucleus were correlated with Siglec-15 expression in SHH-MB patients (Fig. 5G and 5H). In line with these results, Daoy cells highly expressed IDO1, leading to high levels of kynurenine (Kyn) in Daoy xenografts (Fig. 5I and 5J). IDO1 knockdown reduced Kyn levels and abrogated AhR activity; however, this effect was rescued by the addition of exogenous Kyn (Figs. 5L, 5M, S5G, S5I and S5J). Moreover, we found that the tryptophan transporter SLC1A5 was highly expressed in Daoy and primary SHH-MB cells, and knockdown of SLC1A5 also resulted in AhR inactivation, coupled with decreased Kyn levels (Figs. 5K–M, S5F and S5H–J). Together, these results suggest that AhR transcriptionally regulates Siglec-15 expression.

### Targeting AhR effectively treats SHH-MB *in vivo*

**Figure 6.**
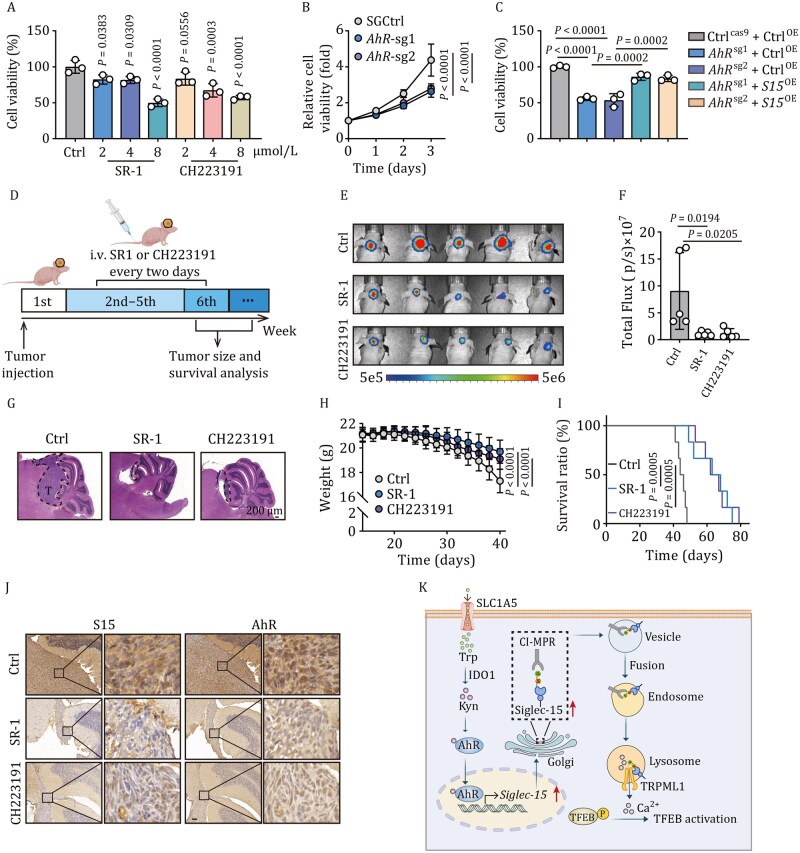
**AhR is a target for SHH-MB treatment**. (A) Cell viability in Daoy cells cultured with Kyn, SR-1, or CH223191 for 24 h was analyzed. (B) Relative cell viability in SG-Ctrl and *AhR*-SG Daoy cells was analyzed. (C) Cell viability in SG-Ctrl, *AhR*-SG, *AhR*-SG-*Siglec-15*-flag Daoy cells was analyzed. (D) Schematic representations of animal experiments. Daoy cells (5 × 10^5^ cells) were orthotopically injected into mice. Mice were treated with SR1 or CH-223191 (10 mg per kg) by intravenous injection every 2 days after 1 week of inoculation. (E and F) Tumor formation was obtained by bioluminescence imaging (E), and total flux was analyzed at 6 weeks (F). (G) Mice were sacrificed, and tumor sizes were evaluated by H&E staining. Tumors in tissues were shown outlined by dashed black lines. T, tumor; *n *= 5 mice. Scale bar, 200 µm. (H and I) Mice weight (H) and survival ratio (I) were analyzed; *n *= 5 mice for (H), *n *= 6 mice for (I). (J) Immunohistochemical staining of Siglec-15 and AhR in Daoy-bearing mice tissues. Scale bar, 200 µm. (K) Schematic diagram of AhR-Siglec-15-TFEB axis mediating sonic hedgehog medulloblastoma growth. AhR directly regulated Siglec-15 expression, and expressed Siglec-15 was transported from the Golgi apparatus to lysosomes. In (A–C), *n *= 3 independent experiments. One-way ANOVA with Dunnett’s multiple-comparisons test (A and F), Tukey’s multiple comparisons test (C), two-way ANOVA with Dunnett’s multiple-comparisons test (B and H), or log-rank survival analysis (I). The data represent mean ± SD.

Finally, we investigated whether targeting AhR could achieve therapeutic efficacy. To this end, we treated Daoy cells with the AhR inhibitor SR1 or CH-223191, and found that cell viability was markedly impaired (Fig. 6A). Such reduced cell viability was also observed in AhR-knockout Daoy cells (Fig. 6B), but was rescued by Siglec-15 overexpression (Fig. 6C). A similar result was obtained from primary tumor cells of SHH-patients (Fig. S6A and S6B). To validate these results *in vivo*, we established luciferase-expressing Daoy MB xenografts. Intravenous administration of SR1 or CH-223191 every 2 days, 1 week after intracranial injection (Fig. 6D), we found that AhR inhibition retarded tumor growth, as evidenced by bioluminescence radiance and H&E staining (Fig. 6E–G), concomitant with increased mouse weight and prolonged survival (Fig. 6H and 6I). In line with this, decreases in Siglec-15 expression and reduced entry of AhR into the nucleus were observed in the treated tumors (Fig. 6J). As expected, AhR knockout also resulted in inhibition of tumor growth (Fig. S6C–F). Intriguingly, forced overexpression of Siglec-15 counteracted the treatment effect of SR1 or CH-223191 on tumor growth in the brain (Fig. S6G and S6H). Together, these results suggest that targeting AhR results in the inhibition of SHH-MB growth by regulating Siglec-15 expression.

## Discussion

Siglec-15 is thought to act as an immune checkpoint and a potential target for cancer immunotherapy. In this study, we unexpectedly revealed the tumor-promoting effect of Siglec-15 in SHH-MB beyond its immune regulation. We found that AhR, Siglec-15, and TRPML1 formed an axis that promoted SHH-MB development. Similar to common secretory proteins, Siglec-15 uses Golgi machinery for transport. However, the upregulation of Siglec-15 by activated AhR and the increased M6P modification resulted in CI-MPR-mediated translocation of Siglec-15 from the Golgi to lysosomal membranes, where Siglec-15 interacts with TRPML1 to initiate the release of lysosomal Ca^2+^ into the cytoplasm. This series of molecular events ultimately triggers the activation of Ca^2+^ signaling and the transcription factor TFEB, leading to the promotion of SHH-MB growth (Fig. 6K).

Mounting evidence has demonstrated that AhR is of paramount importance in cancer biology and immunology (Liu et al., 2017, 2018a; Takenaka et al., 2019). The maintenance of AhR activity in SHH-MB requires active tryptophan (Trp) metabolism. In the cytoplasm, Trp can be catalyzed by IDO1 into Kyn, an endogenous ligand that activates AhR. In line with this, IDO1 was highly expressed in SHH-MB malignant cells. The Trp transporter SLC1A5 was also highly expressed, thus replenishing intracellular tryptophan consumption via active transportation. As a critical cytosolic transcription factor, AhR acts as an exposome receptor that maintains cellular homeostasis by detoxification of xenobiotics (Suzuki et al., 2020). Previously, we found that activated AhR may induce tumor-repopulating cells (TRCs) into a dormant state (Liu et al., 2017). The underlying explanation is that cells use dormancy to counteract sublethal invaders and wake up after spontaneous decay of the invader within the cell. Contradictorily, in this study, we found that activated AhR promoted SHH-MB cell growth rather than dormancy. This may be explained by the fact that rapid cell growth and proliferation may also dilute toxins or invaders. The key question is, what is the switch point that guides cells to enter a dormant or proliferating state? AhR exerts its detoxifying function by transactivating cytochrome P450s (Suzuki et al., 2020). As heme-containing monooxygenases, P450 enzymes catalyze the incorporation of one oxygen atom from molecular oxygen into the toxic molecule RH, yielding innoxious ROH and reactive oxygen species (ROS) as byproducts. However, our recent studies have demonstrated that AhR acts as a ROS sensor by regulating glycogenolysis and the pentose phosphate pathway (Zhou et al., 2023). In the present study, we found that SHH-MB cells expressed higher levels of ROS than other tumor cell lines. This may explain how AhR selectively regulates Siglec-15 to promote SHH-MB growth. Consistently, it has been reported that elevated ROS levels can activate TRPML1, leading to calcium release and enhanced TFEB nuclear translocation (Zhang et al., 2016), providing further insights into the role of the Siglec-15 and TRPML1 combination in regulating TFEB and promoting tumor growth. A deeper understanding of the role of ROS in the AhR-Siglec-15-TRPML1-TFEB axis warrants further investigation.

Evidence further suggests that Siglec-15 is predominantly localized in the lysosome. As a metabolic signaling center, lysosomes possess the ability to detect the presence of nutrients and growth factors, thereby regulating cellular metabolism (Jewell et al., 2013; Li et al., 2019; Medina et al., 2015). In this study, we demonstrated that the interaction between Siglec-15 and TRPML1 leads to calcium release, subsequently activating TFEB. Given that lysosomes serve as an intracellular reservoir for calcium, they play a crucial role in maintaining Ca^2+^ homeostasis and signal transduction (Medina and Ballabio, 2015; Medina et al., 2015). Previous studies have pointed out that TFEB transcriptionally activates the lysosome autophagic pathway during starvation (Medina et al., 2015). Thus, our results provide a novel effect of TRPML1 release calcium trigger TFEB dephosphorylation and nuclear translocation. It is important to note that lysosomal proteins originate from two sources: endocytosis and the autophagic pathway (Saftig and Klumperman, 2009; Yu et al., 2018). The classic lysosomal localization label is mannose 6-phosphate (M6P) (Ghosh et al., 2003). This requires a specific sequence (Asn-X-Ser/Thr) in the protein, which is produced by continuous processing and modification of the ER and the Golgi apparatus (Bonifacino and Traub, 2003). Evidence showed that Siglec-15 is expressed not only on the cell membrane but also in the cytoplasm; however, blocking the endocytosis pathway of Siglec-15 does not affect its localization on the lysosome. In line with this, we demonstrated that Siglec-15 binds to CI-MPR through its N-glycosylation binding site N172. Both N172Q mutation and CI-MPR knockdown resulted in the reduction of Siglec-15 localization to the lysosome and of the promoting effect on tumor growth without the alteration of Siglec-15 on the plasma membrane, suggesting that the Siglec-15 on lysosomes rather than on the plasma membrane promotes cell proliferation. Therefore, inhibition of Siglec-15 lysosomal transport pathway can block MB development, which also provides a new idea for tumor-targeted therapy.

In summary, the data in the present study revealed that AhR activation promotes Siglec-15 transcription by binding to TRPML1 on the lysosome after being transported by CI-MPR. Siglec-15 promotes Ca^2+^ release, which leads to TFEB dephosphorylation and nuclear translocation, thereby promoting SHH-MB growth. The potential therapeutic implications of AhR-Siglec-15 axis modulation in MB cannot be overlooked. Targeted therapies may offer new avenues for treatment, either as standalone interventions or in combination with existing therapies.

## Supplementary Material

pwaf100_Supplementary_Data

## Data Availability

All data needed to evaluate the conclusions in the paper are present in the paper or the Supplementary Materials. Materials described in the study are either commercially available or available upon request from the corresponding author.

## Abbreviations

AhR, aryl hydrocarbon receptor; CI-MPR, cation-independent mannose 6-phosphate receptor; ER, endoplasmic reticulum; GLI, glioma-associated oncogene; GPN, Glycyl-L-phenylalanine 2-naphthylamide; GST, glutathione S-transferase; P2X4, purinergic receptor X4; PTCH1, patched 1; SHH-MB, Sonic hedgehog subgroup medulloblastoma; Siglec-15, sialic acid-binding immunoglobulin-like receptor 15; SMO, smoothened; SR1, StemRegenin 1; SUFU, suppressor of fused; TFEB, transcription factor EB; TGN, trans-Golgi network; TRPML1, mucolipin TRP cation channel 1; ROS, reactive oxygen species; XRE, xenobiotic response element.
