# Supplementary material for: AhR-Siglec-15 axis regulates lysosomal Ca2+ release for sonic hedgehog medulloblastoma growth via TRPML1
Source: Protein Cell. 2025 Nov 19;17(5):438–51. doi: 10.1093/procel/pwaf100 (PMC13161473; doi:10.1093/procel/pwaf100)
Supplement: pwaf100_Supplementary_Data [file pwaf100_supplementary_data.pdf]

Materials and Methods

Animals and cell lines

4- to 6-week-old male BALB/c nude mice were purchased from the Center of Medical Experimental Animals of the Chinese Academy of Medical Science (Beijing, China). All the animals were maintained in the Animal Facilities of Chinese Academy of Medical Science under specific pathogen-free conditions. All studies involving mice were approved by the Animal Care and Use Committee of Chinese Academy of Medical Science.

Human medulloblastoma cancer cell lines Daoy, ONS-76 and human glioblastoma cell line U87 were purchased from China Center for Type Culture Collection (Beijing, China). Daoy, ONS-76 and U87 were cultured in DMEM with 10% FBS and 1% penicillin/streptomycin. Cells were tested for mycoplasma detection and inter-species cross-contamination, and were authenticated by isoenzyme and short-tandem repeat (STR) analyses in the Cell Resource Centre of Peking Union Medical College before the study.

Primary tumor cells isolation

Primary tumor cells were dissociated from the brain tissue of human SHH-MB patients. The fresh tissue specimens were washed with PBS twice and cut into ~1mm^3 pieces on ice, resuspended in 10 ml NS-A basal medium (05750, STEMCELL Technologies) containing 1% penicillin/streptomycin, 0.5 mg/ml DNase I (10104159001, Roche), 0.5 mg/ml Dispase II (04942078001, Roche) and 0.5 mg/ml collagenase IV (C5138, Sigma-Aldrich) and incubated at 37°C for 20 min followed by gentle pipetting. The tissue dissociation solution was filtered through a 100 μm cell strainer (Fisher Scientific, Suwannee, GA, USA) and single cell suspension was collected after lysing red blood cells. Primary tumor cells were cultured in NS-A basal medium with NeuroCult^{TM} NS-A proliferation supplements. The cultured dishes were pre-coated with 1 mg/ml Laminin (L2020, Sigma-Aldrich) dissolved in PBS without Ca^{2+} and Mg^{2+} for 1 hr at 37°C.

Human specimen approval

All human SHH-MB tumor samples were obtained from the First Affiliated Hospital of Zhengzhou University (Table S2). Ethics permission was granted by the First Affiliated Hospital of Zhengzhou University (2023-KY-0342). All normal cerebellar tissues were provided by National Human Brain Bank for Development and Function, Chinese Academy of Medical Sciences and Peking Union Medical College, Beijing, China (Table S1).

Animal experiments and treatment protocol

For orthotopic injection of tumor cells, mice were anesthetized with isoflurane and placed in stereotaxic apparatus. After exposing the skull with a scalpel, a 1 mm diameter hole was drilled on the skull over the cerebellum using a needle. A cell suspension (5×10^5 cells in 5 μl PBS) was slowly injected into the right side of the cerebellum at the following location: 2 mm posterior from Lambda, 1 mm lateral, 3 mm deep and 1 mm retraction to inject at a depth of 2 mm, using a 10 μl Hamilton Syringe (SYR 10 μl, 701N).

For AhR inhibitors or shh inhibitor treatment, mice were treated with SR1 (S2858, Selleck), CH-223191 (HY-12684, MedChemExpress) by intravenous injection every two days after one week of inoculation. Tumor formation was followed by bioluminescence imaging on IVIS spectrum (PE,1400228S). All studies involving mice were approved by the Animal Care and Use Committee of the Chinese Academy of Medical Science (ACUC-A02-2020-009).

Plasmids

pLV-EF1a-IRES-Puro (#85132), pX459 (#62988), pCDNA3.1-3xFlag (#182494), pET-GST (#42049) and pLKO.1 (#8453) vector plasmid, pMD2. G envelope plasmid (#12259) and psPAX2 packing plasmid (#12260) were all purchased from Addgene (MA, USA). cDNA encoding human TRPML1 (HG12763) was purchased from Sino-Biological. N172Q and R143A site mutation plasmids were constructed using a site-directed mutagenesis kit (FM111-01, TransGen Biotech). All plasmids were verified by DNA sequencing.

To generate truncated forms of Siglec-15, cDNAs encoding full-length, extracellular domain (ED), extracellular and transmembrane domain (ED+TD) of Siglec-15 were generated by PCR and cloned into pCDNA3.1-3×Flag vector under infusion, respectively. Deleted, truncated and point mutants were also generated by PCR-based amplification using a construct coding the wild-type as the template. All plasmids were confirmed by DNA sequencing.

RNA interference

Cells were transfected with siRNA interfering target gene expression. All of the siRNAs were transfected using Lipofectamine^{TM} RNAiMAX Transfection Agent (13778100, Life Technologies, Invitrogen). The siRNA sequences were listed in Table S3.

Generation of knockdown and CRISPR-Cas9 knockout cell lines

The shRNAs were cloned into the pLKO.1 vector plasmid and sgRNAs were cloned into the pX459 vector plasmid, then transfected into 293T cells together with the packing plasmids psPAX2 and pMD2.G. After 48 hrs, the lentivirus was collected and concentrated to infect cells together with polybrene at a final concentration of 8 μg/ml. After 48 hrs, infected cells were treated with puromycin at a final concentration of 2 μg/ml. Puromycin-resistant cells were seeded in 96-well cell plates. The candidate knockout cells were verified by western blot. The shRNA and sgRNA sequences were listed in Table S4.

Stably overexpressing Siglec-15

The cDNA for Siglec-15 was gained by PCR and inserted into the lentiviral vector plasmid pLV-EF1α-IRES-Puro with a carboxy-terminal 3×Flag tag for transient expression in 293T cells to obtain the lentivirus containing the target gene. The lentivirus containing Siglec-15 was transduced with Daoy cells. These infected cells were then cultured with 2 μg/ml puromycin to select cell clones with high expression of target genes. The efficiency of overexpression of target genes was confirmed by western blot.

Nuclear/Cytosol fractionation

Cytoplasmic and nuclear proteins were isolated by use of a Cell Fractionation Kit (K266-25, BioVision) according to the manufacturer's instruction. Briefly, the cytoplasmic fraction was collected by adding cytosol extraction buffer and centrifuged for 5 min at 16000 g at 4℃, with the supernatant fraction being the cytosolic fraction. Nuclear fraction was collected by adding nuclear extraction buffer to the pellet and centrifuged for 5 min at 16000 g at 4℃. Equal cell equivalents were analyzed by western blot.

Lysosome isolation

Lysosomal fractions were extracted by differential centrifugation followed by OptiPrep density centrifugation according to the manufacturer's protocol (Lysosome Isolation Kit, LYSISO1, Sigma-Aldrich). The whole procedure was performed at 4℃. Briefly, cells were collected and resuspended in extraction buffer. Then, cells were broken by homogenizer and homogenates were centrifuged for 20 min at 20000 g. After centrifugation, the supernatant fraction was collected as cytosolic fraction while pellet fraction was subjected to additional OptiPrep density centrifugation for 4 hrs at 150000 g at 4℃. The supernatant fraction was centrifuged for 20 min at 20000 g at 4℃ to pellet lysosomes for western blot.

Lysosome pH determination

Cells were incubated with LysoSensor Yellow / Blue DND-160 (L7545, Thermo Fisher) for 30 min at 37℃. After washing twice with cold PBS, the labeled cells were incubated at 37 ℃ for 5 min with 10 μM monensin and 10 μM nigericin in Living Cell Imaging Solution (A14291DJ, Thermo Fisher). The fluorescent intensity was measured at Ex-330/Em-440 and Ex-380/Em-550. Standard pH value curve was obtained by use of an Intracellular pH Calibration Buffers Kit (P35379, Thermo Fisher) containing a range of pH calibration buffers (pH 4.5, 5.5, 6.5 and 7.5). These were used to resuspend cells which can quantify intracellular pH, with the fluorescence intensity of the probes being an indicator of lysosomal pH.

Determination of intracellular Ca^{2+} concentrations

Cells were incubated with 5 μM Fluo-4 AM (500 μl/well, diluted in D-Hanks buffer) for 30 min at 37 °C. 1 ml of D-Hanks was left in each well after washing three times with D-Hanks solution. Then, 1 μM of ionomycin (HY-13434, MedChemExpress) or 100 μM of GPN (HY-W011063, MedChemExpress) was added and the changes in the intracellular calcium concentration were determined using confocal microscope at 488 nm for 180 s for each group.

Cell viability detection

Cell viability was measured using a CellTiter-Glo® Luminescent Cell Viability Assay Kit (G7570, Promega), which is based on the luciferase reaction to measure the amount of ATP present in viable cells. The amount of ATP in the cells correlates with cell viability.

Cell cycle assays

Cells were incubated with 50 mM BrdU for 2 h, and cell cycle analysis was performed using BD Pharmingen APC-BrdU Flow Kits, according to the manufacturer's protocol (559619, BD Bioscience). The following cell cycle phases were determined as a percentage of the total population: G0/G1 (2n, BrdU-negative),

S (2n–4n, BrdU-positive), and G2/M phase (4n, BrdU-negative).

Cell apoptosis detection

Cell apoptosis was determined by Annexin V/Propidium Iodide (PI) Staining kit, according to the manufacturer's protocol (556547, BD Bioscience).

Luciferase assays

5×10^5 293T cells were transfected with 100 ng Renilla luciferase plasmid (pRL-SV40), 1 µg firefly luciferase plasmid pGL4.10-*Siglec-15* promoter and 1 µg of pCMV-*AhR* plasmid for 12 hrs. The cells were then treated with or without 500 µM Kyn for another 24 hrs. Cell lysates were analyzed using a Dual-Luciferase® Reporter assay (E1910, Promega) on a GloMax Multi Plus. Firefly luciferase activity was normalized to Renilla luciferase.

Western Blot assays

Cells were collected, lysed in RIPA lysis buffer and sonicated. Protein concentrations were determined by a BCA kit (Beyotime, China). Then, protein was run on an SDS-PAGE gel and transferred to nitrocellulose. Nitrocellulose membranes were blocked in 5% bovine serum albumin (BSA) and probed with antibodies overnight: anti-β-actin (Cell Signaling Technology, Cat#3700S; 1:1000); anti-Siglec15 (Thermo Fisher, Cat.PA5-114527; 1:1000); anti-flag (Sigma-Aldrich, Cat.F1804; 1:3000); anti-Lamp1 (Cell Signaling Technology, Cat#9091S; 1:1000); anti-Tubulin (Proteintech, Cat#10094-1-AP; 1:1000); anti-p-S6k (Cell Signaling Technology, Cat.9209S; 1:1000); anti-S6k (Cell Signaling Technology, Cat.9092S; 1:1000); anti-p-4EBP1 (Cell Signaling Technology, Cat.2855S; 1:1000); anti-4EBP1 (Cell Signaling Technology, Cat.9644S; 1:1000); anti-TRPML1(Abcam, Cat.ab272608; 1:1000); anti-p-TFEB (Cell Signaling Technology, Cat.37681S; 1:1000); anti-TFEB (Cell Signaling Technology, Cat.37785S; 1:1000); anti-Histone H3 (Cell Signaling Technology, Cat.4499S; 1:1000); anti-AhR (Cell Signaling Technology, Cat.83200S; 1:1000); anti-IGF2R (Cell Signaling Technology, Cat.14364S; 1:1000); anti-IDO1 (Proteintech, Cat.66528-1-Ig; 1:1000) ; anti-IDO2 (Proteintech, Cat.25053-1-AP; 1:1000); anti-TDO (Proteintech, Cat.15880-1-AP; 1:1000); anti-SLC1A5 (Cell Signaling Technology, Cat.8057S; 1:1000); anti-M6P (Creative Biolabs, Cat. PSBL-276; 1:2000). Secondary antibodies conjugated to horseradish peroxidase were followed by enhanced chemiluminescence (Thermo Fisher). Results were confirmed by at least three independent experiments.

Co-Immunoprecipitation

Briefly, indicated plasmids were transfected into either 293T cells or tumor cells, cells were lysed with IP buffer, sonicated, and centrifuged. The supernatants were subjected to IP by incubating them with anti-Flag antibody for 4 hrs or with indicated antibodies overnight at 4°C before incubating with Protein A/G beads for 4 hrs. The beads were washed five times with IP buffer and the protein complexes were denatured using 6×SDS Loading Buffer for western blot.

PCR with reverse transcription

Total RNA was extracted from cells using Trizol (Invitrogen) and was transcribed to cDNA by using a High-Capacity cDNA Reverse Transcription Kit (Cat. 4368813, Applied Biosystems). The primer sequences are listed in Table S5.

Histology and Immunofluorescence

The tumor tissues obtained from mice or medulloblastoma patients were fixed in 10% formalin, embedded in paraffin and sectioned for H&E staining or immunofluorescence. Cells were fixed in 4 % paraformaldehyde and permeabilized with 0.2 % Triton X-100. Fixed cells were blocked in 5 % BSA and incubated with anti-Siglec15 (Thermo Fisher, Cat.PA5-72765; 1:200); anti-Lamp1 (Cell Signaling Technology, Cat.9091S; 1:200); anti-Rab7 (Abcam, Cat.ab137029; 1:200); anti-Calnexin (Abcam, Cat.ab22595; 1:200); anti-TGN46 (Novus, Cat.NBP1-49643; 1:200); anti-ATP5A (Abcam, Cat.ab14748; 1:200); anti-flag (Sigma-Aldrich, Cat.F1804; 1:200); anti-flag (Abcam, Cat.ab1162; 1:200); anti-TRPML1 (Abcam, Cat.ab272608; 1:200); anti-TFEB (Cell Signaling Technology, Cat.37785S; 1:1000); anti-IGF2R (Novus, Cat.NB300-514; 1:200) and anti-AhR (Genetex, Cat. GTX129013; 1:200) at 4℃ overnight. Then, cells were washed and incubated with secondary antibodies for 1 hr at room temperature. Finally, the slides were counterstained with DAPI and mounted for confocal analysis. Representative images were obtained by Nikon A1 microscope or Super-resolution structured illumination microscope (GE Deltavision OMX SR). Immunofluorescence intensity was analyzed using Image J 9.0 software.

ChIP-qPCR assays

ChIP was performed by using an iDeal ChIP-seq kit for Transcription Factors (C01010055, Diagenode, Belgium) according to the manufacturer’s protocol. In brief, 4×10^6 cells were cross-linked with 1% formaldehyde for 8 min at room temperature. After stopping the fixation with 0.125M glycine for 5 min at room temperature, cells were then washed, lysed, and sheared by sonication using Bioruptor (Diagenode, Belgium) for 30 cycles (30s “ON”, 30s “OFF”) at high power setting. The sheared chromatin fragments were immunoprecipitated with protein A-coated magnetic beads and either anti-AhR (Cell Signaling Technology, Cat.83200S) antibody. After elution and decross-linking, the enriched DNA fragments were purified by IPure beads v2. The primer sequences used for ChIP-qPCR are shown as follows: *SIGLEC15*, 5’-AGGATGGGATGGAAGAAGGG-3’ (sense) and 5’-TGGCTGCCATCCCCTCACA-3’ (antisense). These results were obtained from three independent experiments followed by normalization to input signals and shown as mean ± SD.

Recombinant TRPML1-E1 protein expression and purification

To obtain TRPML1-E1 (residues 87-298) protein, E. coli BL21 (DE3) cells harbouring the TRPML1 plasmid (pET28a-6xHis vector) were grown in LB medium supplemented with 30 μg/mL kanamycin. Protein expression was induced overnight at 16°C with 0.1 mM IPTG after OD600 value of 0.8 was reached. Cells were lysed in buffer containing 20 mM HEPES (pH 7.4), 150 mM NaCl, 1mM DTT, 1mM PMSF, 1 mg/ml lysozyme and 1% Triton X-100. The protein was affinity-purified by Ni-Sepharose beads (GE Healthcare Life Sciences). The untagged protein was further washed by 20 mM imidazole.

GST tag and GST-TRPML1-E1 fusion protein (pET-GST vector) were purified using a GST-tag Protein Purification Kit (Beyotime, P2262) with the BeyoGold™ GST-tag Purification Resin according to the manufacturer’s instructions.

Bio-layer interferometry (BLI)

The bio-layer interferometry assay was performed on the Octet RED96 System (ForteBio) with PBS as a running buffer. The operating temperature was maintained at 30°C. N-terminal Siglec-15 protein (CW37, Novoprotein) at a concentration of 10 μg/ml was biotinylated using the Biotinylation Kit (G-MM-IGT, Genemore) and then loaded onto SSA Biosensors (ForteBio) that had been equilibrated in the running buffer. The biosensors were then incubated with various concentrations of the purified TRPML1-E1 (50, 25, 12.5, 6.25, and 3.125 nM), E2, E3, E4 (10 μM), followed by dissociation phase in the running buffer. Data were analyzed and the binding parameters were determined using the software supplied by the manufacturer.

Pull down

293T cells overexpressing *Siglec-15*-ED-flag were collected, lysed on ice and centrifuged at 15000 rpm for 15 min at 4°C. Purified GST fusion proteins or purified GST as a negative control was incubated with GST-Beads for 1hrs at 4°C. The cell lysate was then added to the mixture and incubated overnight at 4°C. The beads were collected by centrifugation at 1000 g for 30 s at 4°C and washed three times with lysis buffer, and the protein complexes were denatured using 6×SDS Loading Buffer for western blot.

Analysis of Kyn concentrations by high performance liquid chromatography (HPLC)

Kyn determination was performed by Dionex UltiMate3000. 1×10^6 Cells or 30-50 mg tissue were lysed by 80% methanol on ice. Samples were frozen and thawed 3 times and centrifuged, then 10 μl of the supernatant was analyzed by HPLC. Standard curves were generated with L-Kyn in the same conditions. The mobile phase consisted of 15 mM sodium acetate containing 8.0% acetonitrile, pH 4.0, which was pumped at a flow rate of 1.0 ml min^{-1}. The analytical column was a C18 chromatographic column (Thermo, 250 mm × 4.6 mm, 5 μm) and kept at 30 °C. Kyn was measured at wavelengths of 360 nm.

Bioinformatics analysis

Gene expression matrix containing Siglec-15 of MB were retrieved from GEO cohort (GSE124814) comprising 1213 MB cases and 291 normal cerebellar samples, t-test was performed to assess the statistical significance of differences between MB cerebellar samples and normal cerebellar samples according to the R package ggpubr. For survival analysis, Kaplan-Meier survival curves and log-rank tests were used to evaluate the outcomes of patients in the GEO cohort (GSE85217) with different Siglec-15 expressions according to the R package survival.

Single-cell transcriptome data were collected from the GEO cohort (GSE155446), which includes 28 childhood MB samples (1 WNT, 9 SHH, 7 Group 3, and 11 Group 4). The R package "Seurat" (version 5.1.0) was utilized to process the single-cell data. Low-quality cells were discarded based on the following criteria: features < 1000, features > 4000, percent.mt < 5%, or counts < 15,000. Cluster identities were manually labeled according to the marker genes identified, SHH (GLI1, GLI2, SFRP1), WNT (DKK1), Group 3 (NPR3, CRX), Group 4 (KCNA1, GRM8), G3/G4 (IMPG1), lymphocytes (TRAC, CD3E), and microglia (TMEM119, AIF1).

Quantification and Statistical Analysis

All experiments were performed at least three times. Results are expressed as means ± SD as indicated. Comparisons between two unpaired groups were evaluated by two-tailed Student's t test. Variance between groups was tested by one-way analysis of variance (ANOVA), followed by Bonferroni's test. Correlations between groups were determined by Spearman's correlation test. The Kaplan-Meier method was used for survival estimation, and the log-rank test was used for comparisons. The numbers of samples and independent biological experimental repeats are indicated in the Fig. or Fig. legends. The P value of <0.05 was considered statistically significant. The analysis was conducted using GraphPad 8.0 software.

Supplementary Materials for

AhR-Siglec-15 axis regulates lysosomal Ca^{2+} release for sonic hedgehog medulloblastoma growth via TRPML1

Zhenfeng Wang *et al*

Corresponding author: tjhuangbo@hotmail.com

The PDF file includes:

Figs. S1 to S6

Table S1 to S5

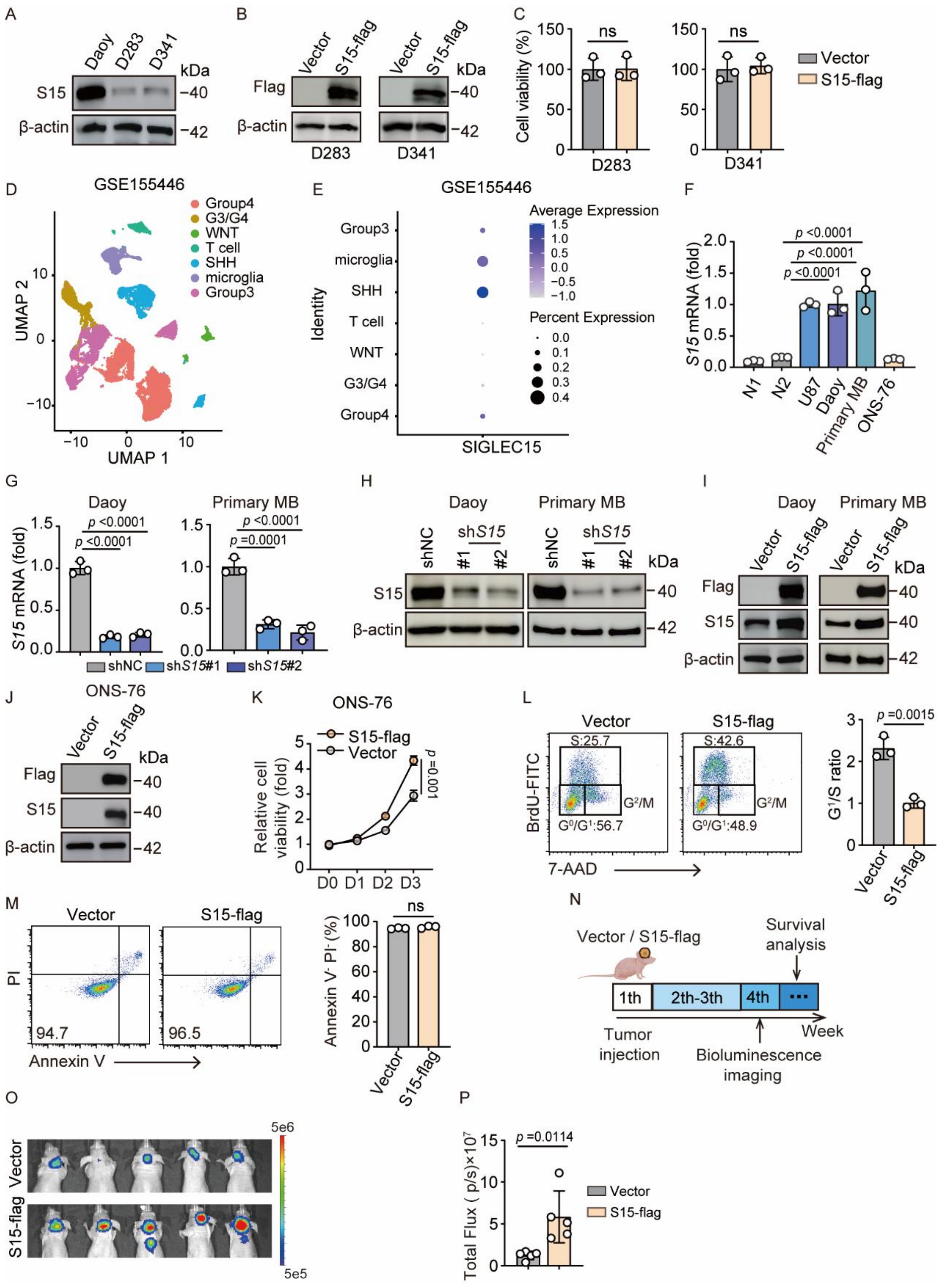

Figure S1. SHH-MB growth required Siglec-15. (**A**) Western blot analysis of Siglec-15 expression in Daoy, D283, and D341 cells. (**B**) Overexpression efficiency of Siglec-15 in D283 and D341 cells was determined by western blot. (**C**) Cell viability in vector or Siglec-15-flag D283 and D341 cells was analyzed. (**D**) Unaligned UMAP projection

shows neoplastic clusters and non-neoplastic clusters. (**E**) Siglec-15 expression in indicated cluster. (**F**) qPCR analysis of Siglec-15 expression in normal human primary granule neuron cells, U87, Daoy, primary tumor cells and ONS-76 cells. S15, Siglec-15. (**G** and **H**) Knockdown efficiency of *Siglec-15* in Daoy and primary tumor cells was determined by qPCR (G) and western blot (H). (**I** and **J**) Overexpression efficiency of Siglec-15 in Daoy, primary tumor cells (**I**) and ONS-76 (**J**) cells was determined by western blot. (**K**) Cell viability in vector or *Siglec-15*-flag ONS-76 cells was analyzed. (**L** and **M**) Cell cycle (L) or cell apoptosis (M) was detected in vector or Siglec-15-flag Daoy cells by flow cytometry. (**N**) Schematic representations of animal experiments. Firefly luciferase-expressing Daoy cells with Siglec-15 overexpression (5×10^5 cells) were orthotopically injected into mice. (**O** and **P**) Tumor formation was obtained by bioluminescence imaging (O), and total flux was analyzed (P) at 4 weeks. n=5 mice. In A-C, F-K, n=3 independent experiments. One-way ANOVA with Dunnett's multiple-comparisons test (**F** and **G**) or two-tailed unpaired Student's t-test (**C, K, L, M** and **P**). ns, not significant. The data represent mean ± s.d.

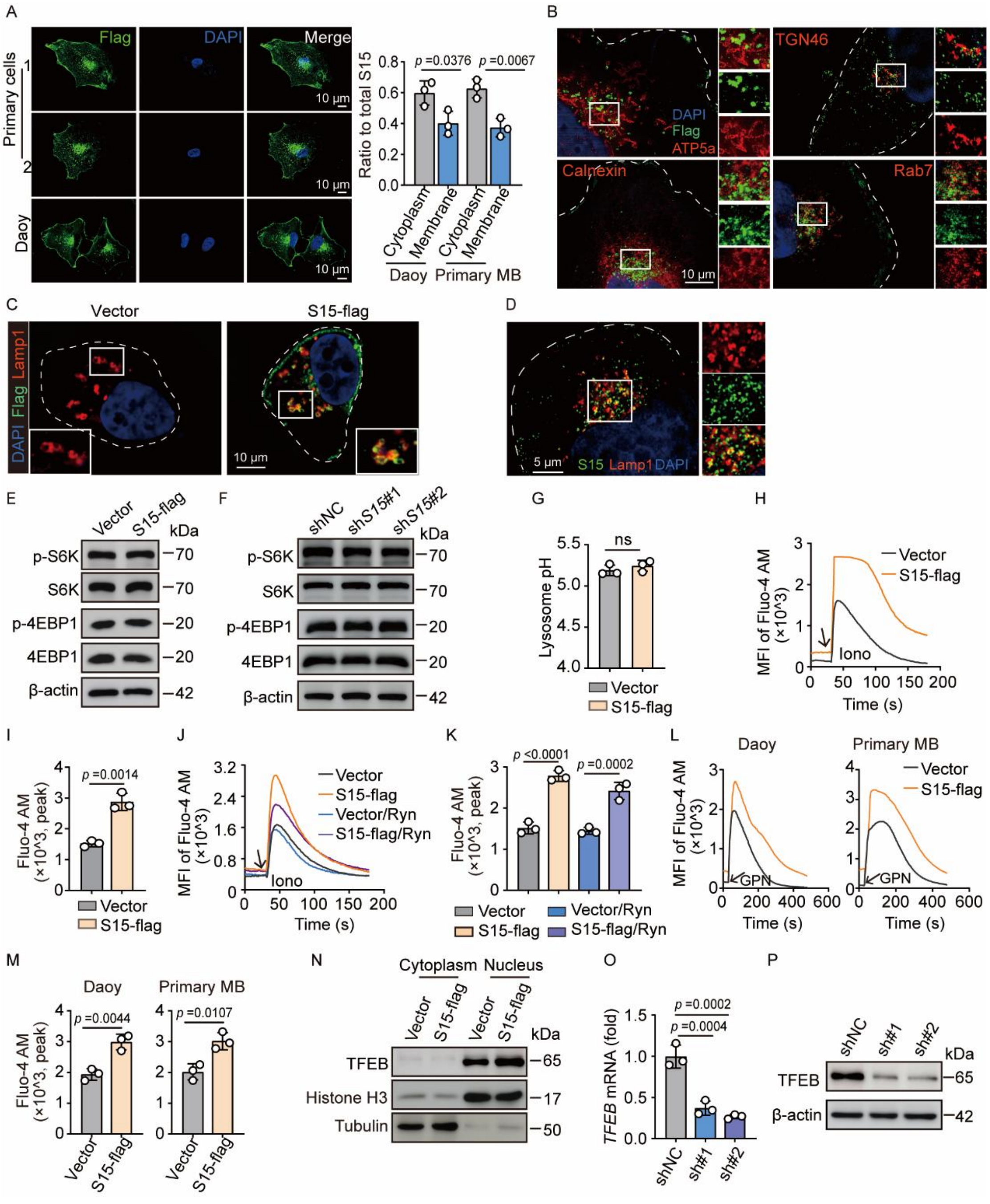

Figure S2. Siglec-15 mediates lysosomal Ca^{2+} release to cytoplasm. (**A**) Immunostaining analysis of *Siglec-15*-flag Daoy cells and primary tumor cells using flag antibody (left). Scale bar, 10 μm. The quantification of Siglec-15 in the cytoplasm and cell membrane by Image J (right). (**B**) Immunostaining analysis of *Siglec-15*-flag Daoy cells using flag, ATP5a, TGN46, Calnexin and Rab7 antibodies. ATP5a, mitochondria marker; TGN46, *trans*-Golgi network marker; Calnexin, endoplasmic

reticulum marker; Rab7, endosome marker. In cellular fluorescence micrographs, representative cells are shown outlined by dashed white lines. Scale bar, 10 μm. (**C**) Immunostaining analysis of *Siglec-15*-flag ONS-76 cells using lamp1 and flag antibodies. Scale bar, 10 μm. (**D**) Immunostaining analysis of Daoy cells using lamp1 and Siglec-15 antibodies. In cellular fluorescence micrographs, representative cells are shown outlined by dashed white lines. Scale bar, 5 μm. (**E**) p-S6K, S6K, p-4EBP1 and 4EBP1 were analyzed by western blot in vector and *Siglec-15*-flag ONS-76 cells. (**F**) p-S6K, S6K, p-4EBP1 and 4EBP1 were analyzed by western blot in sh*Sigelc-15* Daoy cells. (**G**) Lysosomal pH was measured by microplate reader after LysoSensor™ Yellow/Blue DND-160 staining in vector and *Siglec-15*-flag ONS-76 cells. (**H** and **I**) Vector and *Siglec-15*-flag ONS-76 cells were loaded with Fluo-4 AM and cytosolic calcium release was recorded by confocal microscope (H). The peak of Fluo-4 AM was analyzed (I). (**J** and **K**) Vector and *Siglec-15*-flag ONS-76 cells were pretreated with ER calcium release inhibitor ryanodine (10 μM) for 2 hrs and cytosolic calcium release was recorded by confocal microscope (J). The peak of Fluo-4 AM was analyzed (K). (**L** and **M**) Siglec-15-flag Daoy and primary tumor cells were loaded with Fluo-4 AM and cytosolic calcium release was recorded by confocal microscope (L). The peak of Fluo-4 AM was analyzed (M). (**N**) Cytoplasmic and nuclear proteins of vector and *Siglec-15*-flag Daoy cells were isolated, TFEB expression was analyzed by western blot. (**O** and **P**) Knockdown efficiency of *TFEB* was determined by qPCR (O) and western blot (P). In A-P, n=3 independent experiments. ns, not significant, Two-tailed unpaired Student's t-test (**G**, **I**, **M**), one-way ANOVA with Tukey's multiple comparisons test (**K**)

or Dunnett's multiple-comparisons test (**O**). The data represent mean ± s.d.

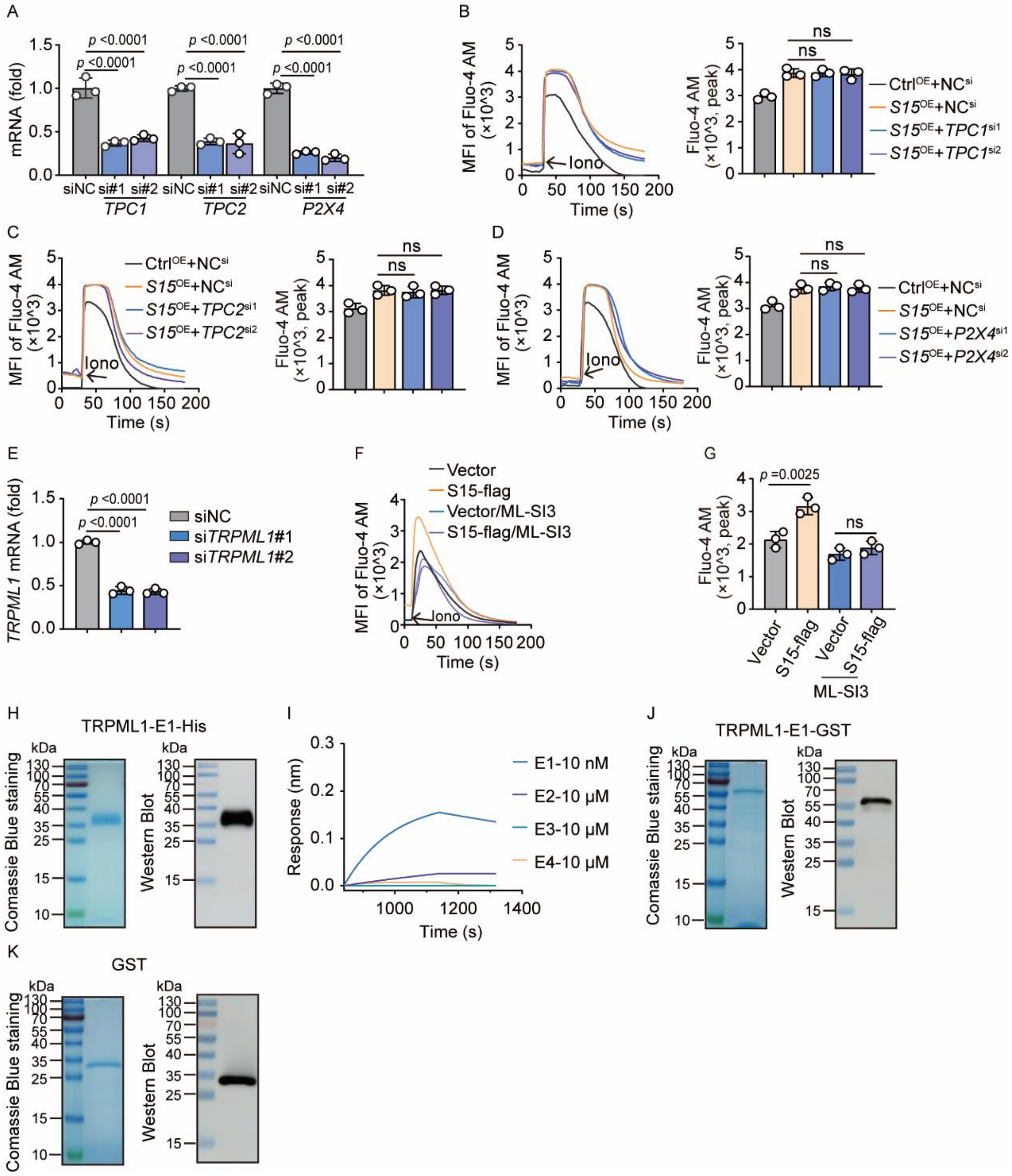

Figure S3. Siglec-15 binds to TRPML1 mediating lysosomal Ca^{2+} release. (**A**) Knockdown efficiency of *TPC1*, *TPC2* and *P2X4* in *Siglec-15*-flag Daoy cells was determined by qPCR. (**B-D**) *Siglec-15*-flag Daoy cells transfected with siNC, si*TPC1*, si*TPC2* and si*P2X4* were loaded with Fluo-4 AM and cytosolic calcium release was recorded using confocal microscope. The peak of Fluo-4 AM was analyzed as shown on the right of B-D. (**E)** Knockdown efficiency of *TRPML1* in *Siglec-15*-flag Daoy cells was determined by qPCR. (**F** and **G**) Vector and Siglec-15-flag Daoy cells were

pretreated with ML-SI3 (10 μM) for 2 hrs, and cytosolic calcium release was recorded by confocal microscope (F). The peak of Fluo-4 AM was analyzed (G). (**H, J, K**) Purified recombinant TRPML1-E1 and GST were verified by Coomassie blue staining and western blot. (**I**) Interaction between recombinant Siglec-15 ED domain and TRPML1 extracellular domain was measured by biolayer interferometry. In A-E, F, I, n=3 independent experiments. One-way ANOVA with Dunnett's multiple-comparisons test (**A**, **E**) or Tukey's multiple comparisons test (**B**-**D** and **G**). The data represent mean ± s.d.

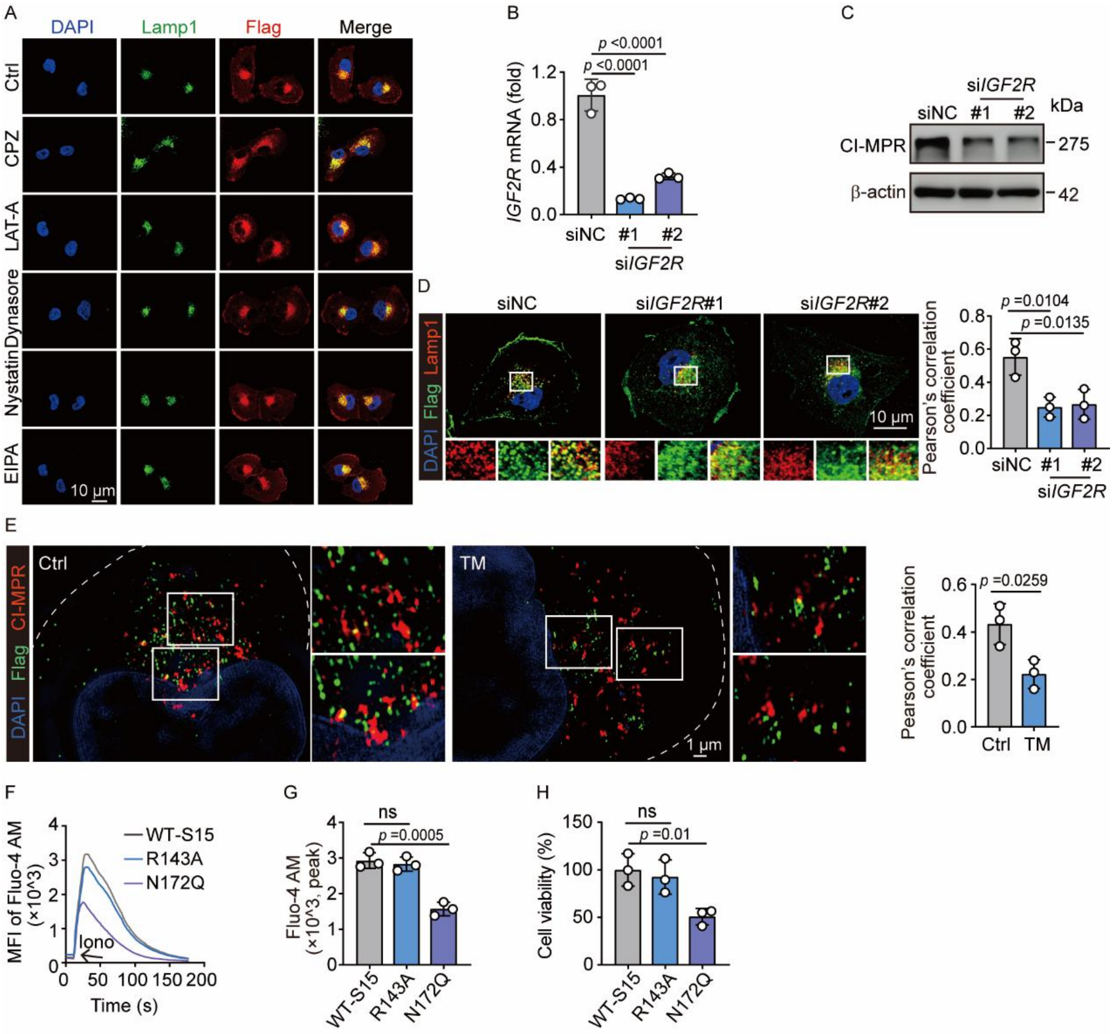

Figure S4. CI-M6PR mediates Siglec-15 transport from the Golgi to lysosomes. (**A**) Immunostaining analysis of lamp1 and flag in *Siglec-15*-flag Daoy cells cultured with 5 μM CPZ, 200 nM LAT-A, 40 μM Dynasore, 10 μM Nystatin, 2.5 μM EIPA for 24 hrs. Scale bar, 10 μm. (**B** and **C**) Knockdown efficiency of *IGF2R* in *Siglec-15*-flag Daoy cells was determined by qPCR (**B**) and western blot (**C**). (**D**) Immunostaining analysis of lamp1 and flag in *Siglec-15*-flag Daoy cells transfected with siNC or si*IGF2R* (left). Scale bar, 10 μm. Pearson's correlation coefficient between flag and lamp1 (right). (**E**) Immunostaining analysis of flag and CI-MPR in Siglec-15-flag Daoy cells pretreated with Tunicamycin (2 μM) or PBS (left). Scale bar, 1 μm. Pearson's correlation coefficient between flag and CI-MPR (right). (**F** and **G**) Daoy cells were loaded with

Fluo-4 AM and cytosolic calcium release was recorded by confocal microscope (**F**). The peak of Fluo-4 AM was analyzed (**G**). (**H**) Cell viability in *Siglec-15*-flag, *Siglec-15*-R143A or N172Q Daoy cells was analyzed. In A-F, H, n=3 independent experiments. One-way ANOVA with Dunnett's multiple-comparisons test (**B**, **G**, **H**). The data represent mean ± s.d.

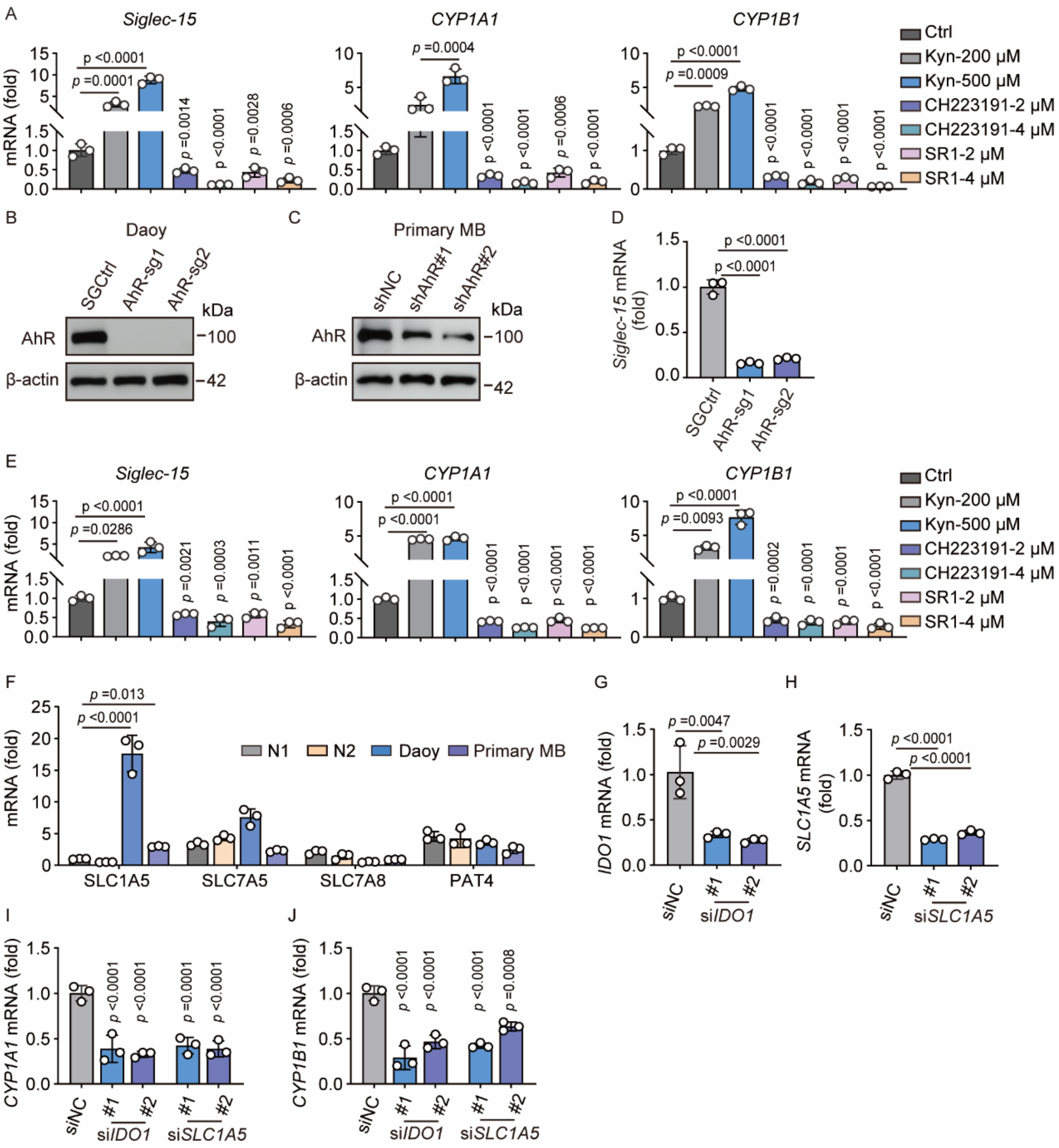

Figure S5. Transcription factor AhR upregulates Siglec-15 expression. (**A** and **E**) qPCR analysis of *Siglec-15*, *CYP1A1* and *CYP1B1* expression in Daoy (A) and primary tumor cells isolated from SHH-MB patients (E) treated with Kyn, CH223191 or SR-1 for 24 hrs. (**B**) Knockout efficiency of *AhR* in Daoy cells was determined by western blot. (**C**) Knockdown efficiency of *AhR* in primary tumor cells was determined by western blot. (**D**) Expression of Siglec-15 in SG-Ctrl and *AhR*-SG Daoy cells was determined by qPCR. (**F**) Expression of *SLC1A5*, *SLC7A5*, *SLC7A8* and *PAT4* in normal human primary granule neuron cells, Daoy and primary tumor cells isolated

from SHH-MB patients was determined by qPCR. (**G** and **H**) Knockdown efficiency of *IDO1* (G) and *SLC1A5* (H) in Daoy cells was determined by qPCR. (**I** and **J**) Expression of *CYP1A1* (I) and *CYP1B1* (J) in siNC, si*SLC1A5* or si*IDO1* Daoy cells was determined by qPCR. In A-J, n=3 independent experiments. One-way ANOVA with Dunnett's multiple-comparisons test (**A**, **D**, **E-J**). The data represent mean ± s.d.

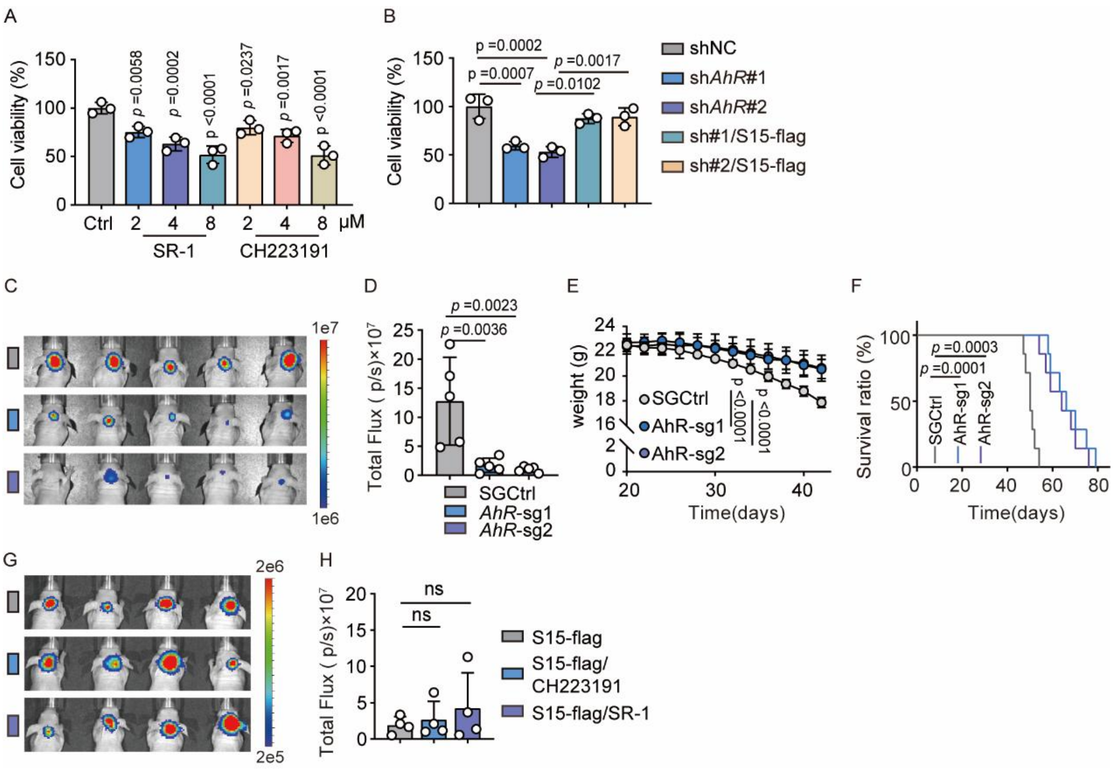

Figure S6. AhR-Siglec-15 axis regulates SHH-MB growth in mice. (**A**) Cell viability in primary tumor cells cultured with Kyn, SR-1 or CH223191 for 24 hrs was analyzed. (**B**) Cell viability in sh-NC, *AhR*-sh, *AhR*-sh-*Siglec-15*-flag primary tumor cells was analyzed. (**C**-**F**) SG-Ctrl and *AhR*-SG Daoy cells (5×10^5 cells) were orthotopically injected into mice. Tumor formation was obtained by bioluminescence imaging at 6 weeks (C) and total flux was analyzed (D) at 6 weeks. Mice weight (E) and survival ratio (F) were analyzed. (n=5 mice for C, D, E, n=7 mice for F). (**G** and **H**) Mice were treated with SR1 or CH-223191 (10 mg per kg) by intravenous injection every two days after one week of *Siglec-15*-flag Daoy cells (5×10^5 cells) inoculation. Tumor formation was obtained using bioluminescence imaging (G) and total flux was analyzed at 4 weeks (H). n=4 mice. In A-B, n=3 independent experiments. One-way ANOVA with Dunnett's multiple-comparisons test (**A**, **D**, **H**), Tukey's multiple comparisons test (**B**), two-way ANOVA with Dunnett's multiple-comparisons test (**E**) or log-rank survival

analysis (**F**). The data represent mean ± s.d.

Table S1. Clinical information of healthy people.

NO.	Gender	Age	Sample
1	F	16	Parafin section and Tumor tissue
2	M	41	Parafin section and Tumor tissue
3	M	24	Parafin section and Tumor tissue
4	M	82	Parafin section and Tumor tissue

Table S2. Clinical information of medulloblastoma patients.

NO.	Gender	Age	Status	Sample
1	F	15	Newly diagnosis (SHH)	Parafin section
2	M	67	Newly diagnosis (SHH)	Parafin section
3	F	13	Newly diagnosis (SHH)	Parafin section
4	M	2	Newly diagnosis (SHH)	Parafin section
5	M	4	Newly diagnosis (SHH)	Parafin section
6	M	16	Newly diagnosis (SHH)	Parafin section
7	F	9	Newly diagnosis (SHH)	Parafin section
8	M	9	Newly diagnosis (SHH)	Parafin section
9	M	27	Newly diagnosis (SHH)	Parafin section
10	F	1	Newly diagnosis (SHH)	Parafin section
11	F	8	Newly diagnosis (SHH)	Parafin section
12	F	6	Newly diagnosis (SHH)	Parafin section
13	M	3	Newly diagnosis (SHH)	Parafin section
14	F	11	Newly diagnosis (SHH)	Parafin section
15	F	10	Newly diagnosis (SHH)	Parafin section

Table S3. siRNA targeting sequence used in this article.

siRNA targeting sequence: *hTRPML1* #1: GAUCACGUUUGACAACAAAGC
siRNA targeting sequence: *hTRPML1* #2: CGGUGCAGCUCAUCCUGUUUG
siRNA targeting sequence: *hIGF2R* #1: GCAUCAAGAUAUCGACUCU
siRNA targeting sequence: *hIGF2R* #2: CCAGCGGCCAGUAUUUCUA
siRNA targeting sequence: *hTPC1* #1: GAAGGCUACUAUUAUCUCA
siRNA targeting sequence: *hTPC1* #2: GCAGAACAUGCGUGCGUUU
siRNA targeting sequence: *hP2X4* #1: GUACUACAGAGACCUGGCU
siRNA targeting sequence: *hP2X4* #2: CCAACAUCACCACUACUUA
siRNA targeting sequence: *hIDO1* #1: GCCUGAUCUCAUAGAGUCU
siRNA targeting sequence: *hIDO1* #2: CAGACGGUCUGGUGUAUGA
siRNA targeting sequence: *hSLC1A5* #1: GUACCGUCCUCAAUGUAGA
siRNA targeting sequence: *hSLC1A5* #2: GAAGCACAGAGCCUGAGUU

Table S4. shRNA and sgRNA targeting sequence used in this article.

shRNA targeting sequence: *h-SIGLEC15* #1:CCGGGGAACATCAAATCCTGGAAACCTCGAGGTTTCCAGGATTTGATGTTCCTTTTTG (sense) and AATTCAAAAAGGAACATCAAATCCTGGAAACCTCGAGGTTTCCAGGATTTGATGTTCC (antisense)
shRNA targeting sequence: *h-SIGLEC15* #2: CCGGGGAATAACGAGGCCATGAACACTCGAGTGTTCATGGCCTCGTTATTCCTTTTTG (sense) and AATTCAAAAAGGAATAACGAGGCCATGAACACTCGAGTGTTCATGGCCTCGTTATTCC (antisense)
shRNA targeting sequence: h-TFEB #1:CCGGCGATGTCCTTGGCTACATCAACTCGAGTTGATGTAGCCAAGGACATCGTTTTTG (sense) and AATTCAAAAACGATGTCCTTGGCTACATCAACTCGAGTTGATGTAGCCAAGGACATCG (antisense)
shRNA targeting sequence: h-TFEB #2: CCGGGGGAGTTGGATGATGTCATTGCTCGAGCAATGACATCATCCAACTCCCTTTTTG (sense) and AATTCAAAAAGGGAGTTGGATGATGTCATTGCTCGAGCAATGACATCATCCAACTCCC (antisense)
sgRNA targeting sequence: *h-AHR* #1:CACCGTCAAGTCAAATCCTTCCAAG (sense) and AAACCTTGGAAGGATTTGACTTGAC (antisense)
sgRNA targeting sequence: *h-AHR* #2: CACCGTTAATAACATCTTGTGGGAA (sense) and AAACTTCCCACAAGATGTTATTAAC (antisense)

Table S5. Primer sequences used in this article.

Primer for qPCR *h-β-actin*: 5'-CCACCCTCTCGGAGATCAAG-3' (sense) and 5'-TCCCTTCGCTTCTGAGTTAGG-3' (antisense)
Primer for qPCR *h-TFEB*: 5'-ACCTGTCCGAGACCTATGGG-3' (sense) and 5'-CGTCCAGACGCATAATGTTGTC-3' (antisense)
Primer for qPCR *h-IGF2R*: 5'-GTGACCAGCAAGGCACAAATC-3' (sense) and 5'-CACCAAGTAGGCACCACTAAG-3' (antisense)
Primer for qPCR *h-TRPML1*: 5'-TTCGCCGTCGTCTCAAATACT-3' (sense) and 5'-CTCTTCCCGGAATGTCACAGC-3' (antisense)
Primer for qPCR *h-Siglec-15*: 5'-CAGCACCGAGATGTTGACGA-3' (sense) and 5'-ACGATCGCTATGAGAGTCGC-3' (antisense)
Primer for qPCR *h-TPC1*: 5'-GGGAGATGAATTACCAAGAGGC-3' (sense) and 5'-GTGGCGTGGACATAGATGCC-3' (antisense)
Primer for qPCR *h-TPC2*: 5'-TACCGCAGCATCCAAGTCG-3' (sense) and 5'-CACCCGGTGGTTGATGGAG-3' (antisense)
Primer for qPCR *h-P2X4*: 5'-CTACCAGGAAACTGACTCCGT-3' (sense) and 5'-GGTATCACATAATCCGCCACAT-3' (antisense)
Primer for qPCR *h-IDO1*: 5'-TCTCACAGACCACAAGTCACAG-3' (sense) and 5'-TGGCAAGACCTTACGGACATC-3' (antisense)
Primer for qPCR *h-SLC1A5*: 5'-TCATGTGGTACGCCCCTGT-3' (sense) and 5'-GCGGGCAAAGAGTAAACCCA-3' (antisense)
Primer for qPCR *h-SLC7A5*: 5'-GGAAGGGTGATGTGTCCAATC-3' (sense) and 5'-TAATGCCAGCACAATGTTCCC-3' (antisense)
Primer for qPCR *h-SLC7A8*: 5'-AGGCTGGAACTTTCTGAATTACG-3' (sense) and 5'-ACATAAGCGACATTGGCAAAGA-3' (antisense)
Primer for qPCR *h-PAT4*: 5'-CGCGAGGAGCTAGATATGGAT-3' (sense) and 5'-TGGAAGTCCTAAAAGGCCAGT-3' (antisense)
